# Neuronal RBM5 modulates cell signaling responses to traumatic and hypoxic-ischemic injury in a sex-dependent manner

**DOI:** 10.1038/s41420-023-01677-7

**Published:** 2023-10-17

**Authors:** Kara Snyder, Kiersten Gorse, Patrick M. Kochanek, Travis C. Jackson

**Affiliations:** 1 University of South Florida, Morsani College of Medicine, USF Health Heart Institute, MDD 0630, 560 Channelside Dr, Tampa, FL 33602 USA; 2 University of South Florida, Morsani College of Medicine, Department of Molecular Pharmacology & Physiology, 12901 Bruce B Downs Blvd, Tampa, FL 33612 USA; 3 Safar Center for Resuscitation Research, UPMC Children’s Hospital of Pittsburgh, Rangos Research Center – 6th floor, Pittsburgh, PA 15224 USA

**Keywords:** Experimental models of disease, Cell death in the nervous system

## Abstract

It is not clear if inhibiting the pro-death gene RNA binding motif 5 (RBM5) is neuroprotective in isolated primary neurons or if it regulates cell survival in a sex-dependent manner. Here we established sex-dichotomized primary cortical neuron cultures from transgenic mice harboring a floxed RBM5 gene-trap. Lentivirus-mediated expression of CRE was used to silence RBM5 expression. Male and female neurons were maintained in next-generation Neurobasal-Plus media and subjected to a mechanical stretch-injury (to model traumatic brain injury) or oxygen-glucose deprivation/OGD (to model ischemia). RBM5 KO did not affect 24 h post-injury survival as determined by lactate dehydrogenase (LDH) release, in either paradigm. In contrast, female KO neurons had increased spectrin breakdown products post-insult (in both models). Furthermore, in OGD, RBM5 KO in male neurons exacerbated injury-induced downregulation of pro-survival AKT activation (pAKT473) but conversely led to pAKT473 sparing in female neurons. Moreover, global proteomics identified 19 differentially expressed (DE) proteins in OGD-injured male neurons, and 102 DE proteins in injured female neurons. Two novel RBM5-regulated proteins (PIGQ and EST1C) were identified in injured male KO neurons, and 8 novel proteins identified in injured female KO neurons (S35A5, DHTK1, STX3, IF3M, RN167, K1C14, DYHS, and MED13). In summary, RBM5 inhibition does not modify neuronal survival in primary mouse neurons in 2 clinically relevant models of excitotoxic insult, but RBM5 does regulate intracellular responses to injury in a sex-dependent manner.

## Introduction

RNA binding motif 5 (RBM5) is a pro-death tumor suppressor gene that promotes apoptosis in cancer cells [1–4]. In brain, it is mainly found in neurons [5]. RBM5 overexpression in rat cortical neurons increased cell death after stretch-injury [6]. Moreover, RBM5 knockdown in human neuronal cells decreased caspase activation in the staurosporine model of apoptosis [5]. Thus, RBM5 inhibition may be neuroprotective [1].

Conversely, the only study to test if RBM5 gene knockout (KO) is neuroprotective in the brain did not observe long-term histological benefits after a traumatic brain injury (TBI) [7]. It is unclear if the lack of protection could have resulted from: (a) detrimental physiological changes in vivo, (b) early benefits being overlooked (i.e., only 7d outcome was investigated), or (c) the insult modality (i.e., RBM5 inhibition has not been explored in non-traumatic injuries like hypoxia-ischemia) [8]. Here we addressed these questions by testing if RBM5 KO is neuroprotective acutely (24 h post-injury) in dissociated primary neurons in vitro, and in two clinically relevant models of neuronal death (TBI-relevant mechanical stretch-injury and hypoxia-ischemia-relevant oxygen-glucose deprivation/OGD) [6, 9–11]. Experiments were also done in next-generation culture media (Neurobasal-Plus/B27-Plus) which supports neuronal properties mimicking the intact brain but are underexplored reagents in brain injury research [6, 12, 13].

RBM5-mediated regulation of cellular processes may be sex-dependent. RBM5 inhibition in mice increased apoptosis in reproductive tissues only in males [14]. Furthermore, we reported that post-TBI levels of α-II-spectrin breakdown products (SBDPs) and phosphorylated CREB (pCREB) are augmented in RBM5 KOs only in the female brain [7]. It is unclear if signaling changes are a direct consequence of RBM5 inhibition in neurons or caused by an indirect effect of RBM5 KO on the brain. However, estrogen receptor alpha (ERα) levels are increased in the hippocampus in non-ovariectomized female KOs after a TBI [7]. Thus, indirect effects involving estrogen signaling could play a role.

Here we report that RBM5 KO did not affect 24 h neuronal survival after either stretch-injury or OGD and was not modified by 17β-estradiol (E2). However, RBM5 modulated neuronal signaling post-injury in a sex-dependent manner. Thus, RBM5 may have distinct functions in neurons in the injured male versus female brain, and which may contribute to the molecular basis mediating sex-differences after a CNS insult.

## Results

### Optimizing mechanical stretch injury to study cell signaling in mouse neurons maintained in next-generation neurobasal/B27-plus media

**Fig. 1:**
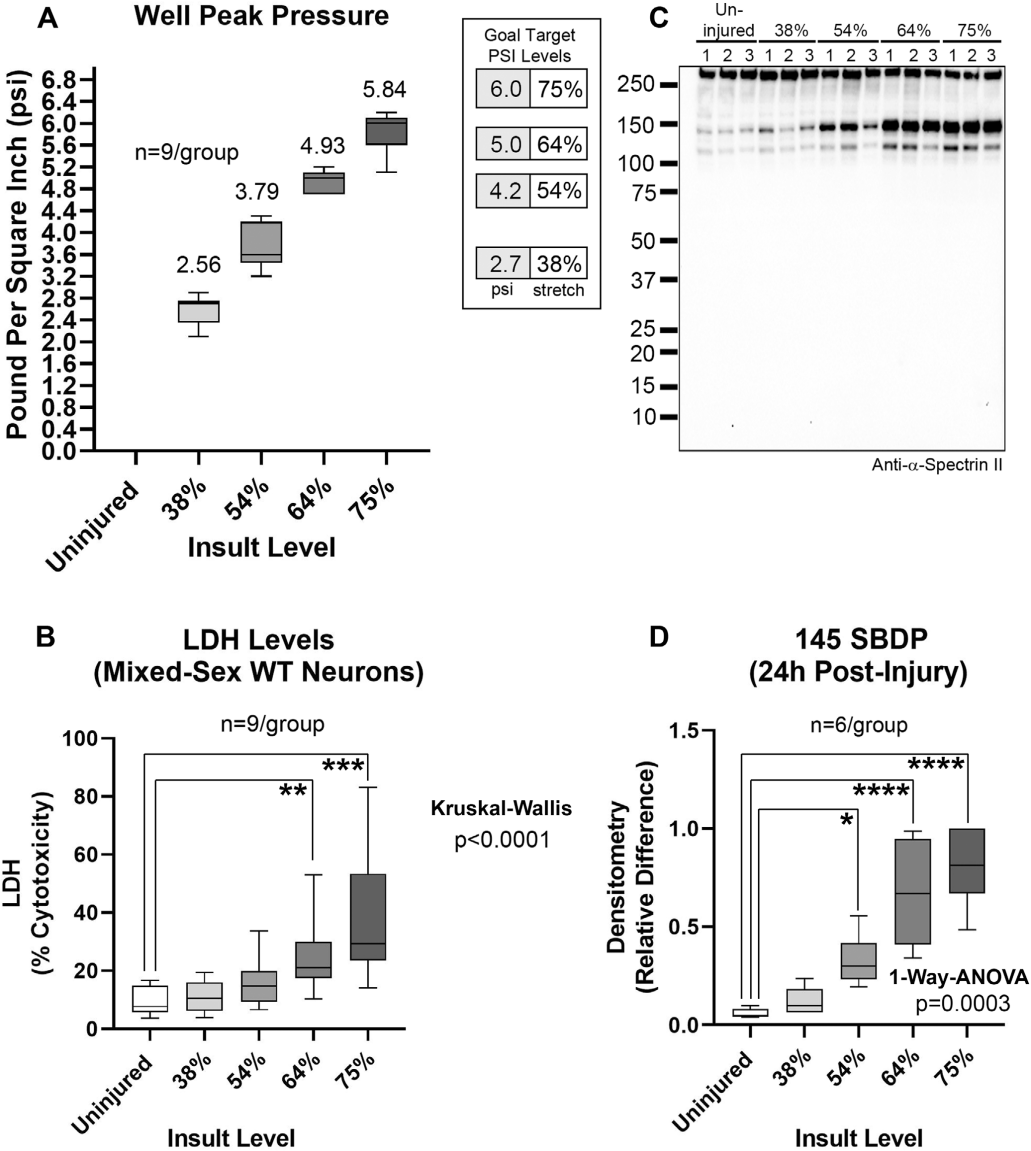
Stretch-injury insult severity in WT mixed-sex mouse cortical neurons in next-generation neurobasal-plus media. **A** Box plots and mean of well peak pressures (psi). The legend indicates the % stretch per psi. **B** Box plots of post-injury LDH levels from 3 independent culture isolations. **C** Representative blot (*n* = 3/group) of SBDP levels. **D** Densitometric quantification (*n* = 6/group) of SBDP 145 levels normalized to total protein stain. Box plots show median, max, min, and IQR. Data were significant at *p* < 0.05. *p < 0.05, **p < 0.01, ***p < 0.001, and ****p < 0.0001. Spectrin breakdown products (SBDPs).

We characterized a range of stretch-injury severities in mixed-sex WT mouse cortical neurons in next-generation Neurobasal-Plus/B27-Plus media (Fig. 1A). The % stretch was associated with 24 h post-injury LDH and SBDP levels (Figs. 1B–D and S1). An intermediate insult severity (64% stretch) was chosen for downstream KO studies.

**Fig. 2:**
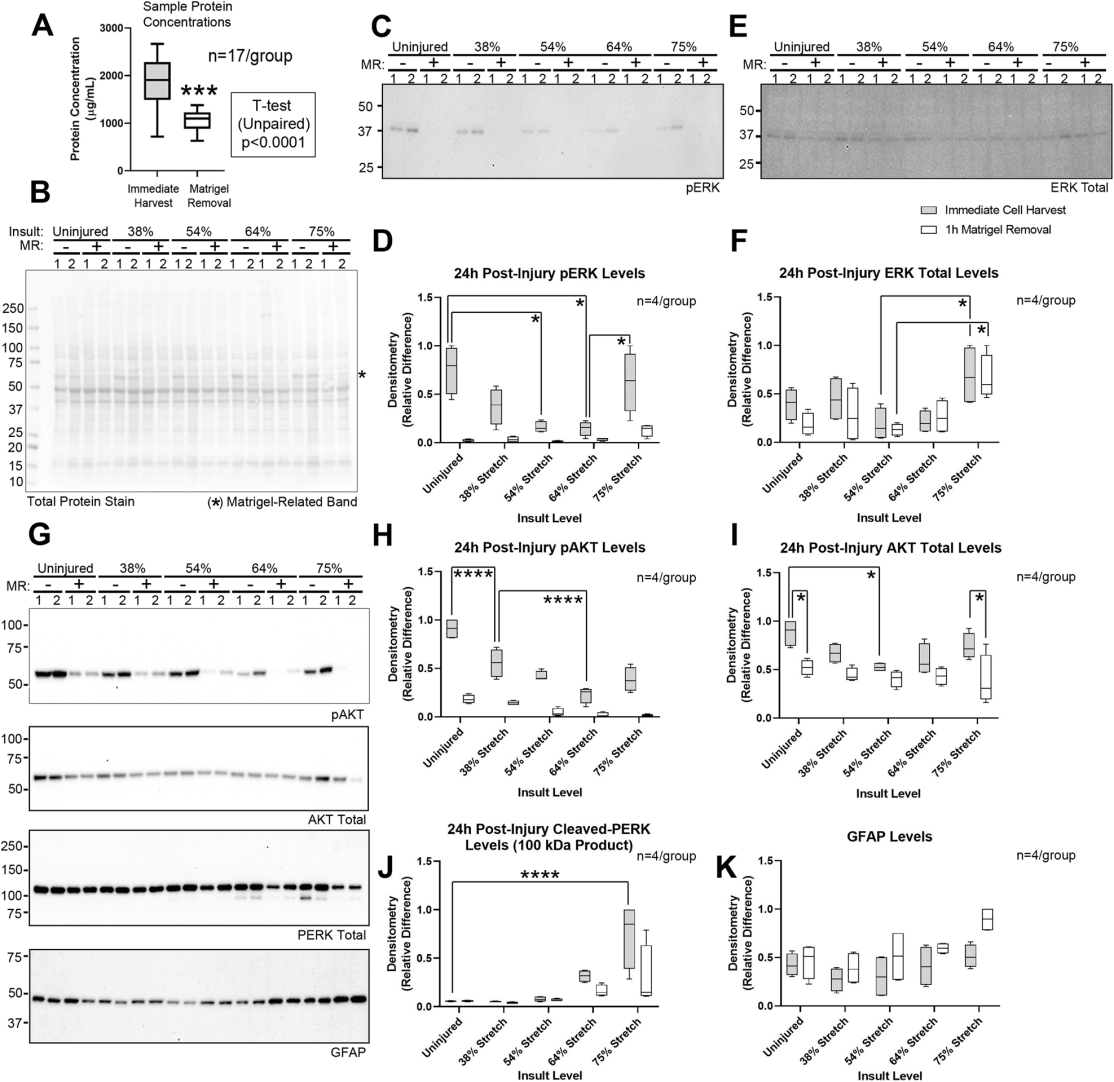
Optimization of sample collection for signaling studies in the stretch model. **A** Total protein concentrations with or without Matrigel removal (MR). **B** Total protein stain for phosphorylated ERK (pERK) in panel 2 C, in samples processed with (+) or without (−) the MR step (*n* = 2/group). The asterisk denotes *a* ~ 60 kDa Matrigel-derived band. **C** Representative blot of pERK levels (*n* = 2/group). **D** Densitometric quantification of pERK normalized to total protein stain from 2 independent culture isolations (*n* = 4/group). **E** Representative blot of total ERK levels (*n* = 2/group). **F** Densitometric quantification of total ERK normalized to total protein stain (*n* = 4/group). **G** Representative blots of phosphorylated AKT (pAKT), total AKT, PERK, and GFAP levels (*n* = 2/group). **H**–**K** Densitometric quantification of pAKT, total AKT, cleaved PERK, and GFAP normalized to total protein stain (*n* = 4/group). Box plots show median, max, min, and IQR. Data were significant at *p* < 0.05. *p < 0.05, ***p < 0.001, and ****p < 0.0001. AKT protein kinase B, ERK extracellular signal-regulated kinase, PERK PRKR-like endoplasmic reticulum kinase, GFAP glial fibrillary acidic protein.

Matrigel^®^ (a mixture of laminins, collagen IV, entactin, and perlecan) increases neuron adhesion to the Silastic membrane and can be dissolved with *Cell Recovery Solution* [6]. However, Matrigel removal (MR) may impede cell signaling studies. MR decreased the amount of protein recovered by ~42% (Fig. 2A). However, total protein staining of PVDF membranes showed similar staining characteristics, except for *a* ~ 60 kDa band in samples containing residual Matrigel (Fig. 2B). Thus, MR likely increases loss of neuron-derived proteins, possibly due to inadvertent cell lysis. Omitting the MR step permitted the detection of phosphorylated pERK and pAKT, revealed incrementally decreased phosphorylation with increasing insult severity, and enhanced detection of non-phosphorylated targets including PERK cleavage products (Figs. 2C–K, S2, S3, and S14 and Table S1).

### Investigating the effect of RBM5 KO on neuronal survival in isolated male and female cortical neurons after a mechanical stretch or OGD injury

**Fig. 3:**
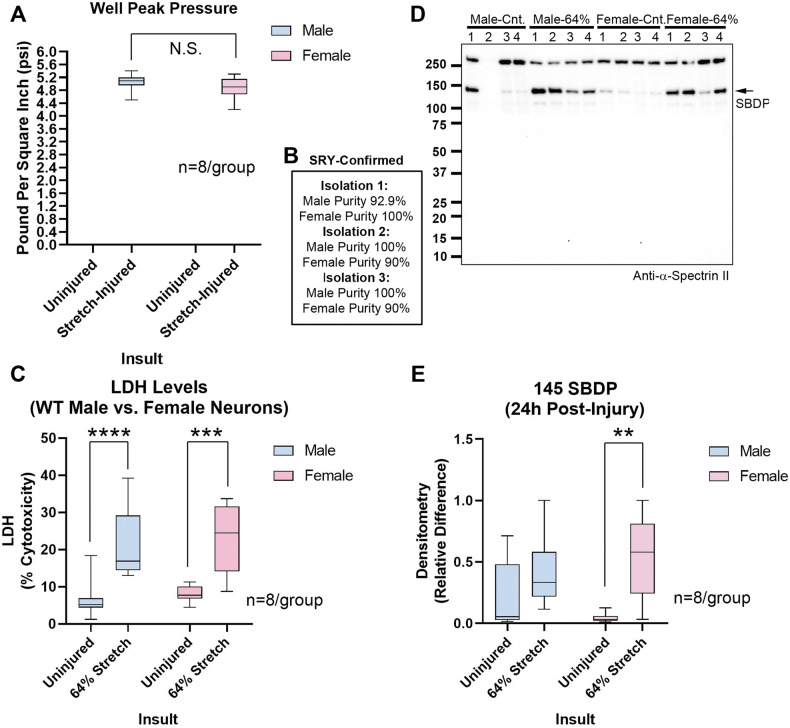
Stretch-injury in sex-dichotomized WT neurons. **A** Box plots show well peak pressure. **B** Purity of male and female cultures in independent isolations in Fig. 3. **C** Box plots show post-injury LDH levels (*n* = 8/group). **D** Representative blot of SBDPs (*n* = 4/group). **E** Densitometric quantification of SBDP normalized to total protein stain (*n* = 8/group). Box plots show median, max, min, and IQR. Data were significant at *p* < 0.05. **p < 0.01, ***p < 0.001, and ****p < 0.0001. N.S. not significant. Spectrin breakdown products (SBDPs).

We next characterized the effect of sex on stretch-injured WT neurons in Neurobasal-Plus. Insult severity (~64% stretch) was equivalent in male and female WT neurons (Fig. 3A, B, and Table S2). No sex differences were observed on 24 h post-injury LDH or SBDP levels (Figs. 3C–E, S4, and Table S2).

**Fig. 4:**
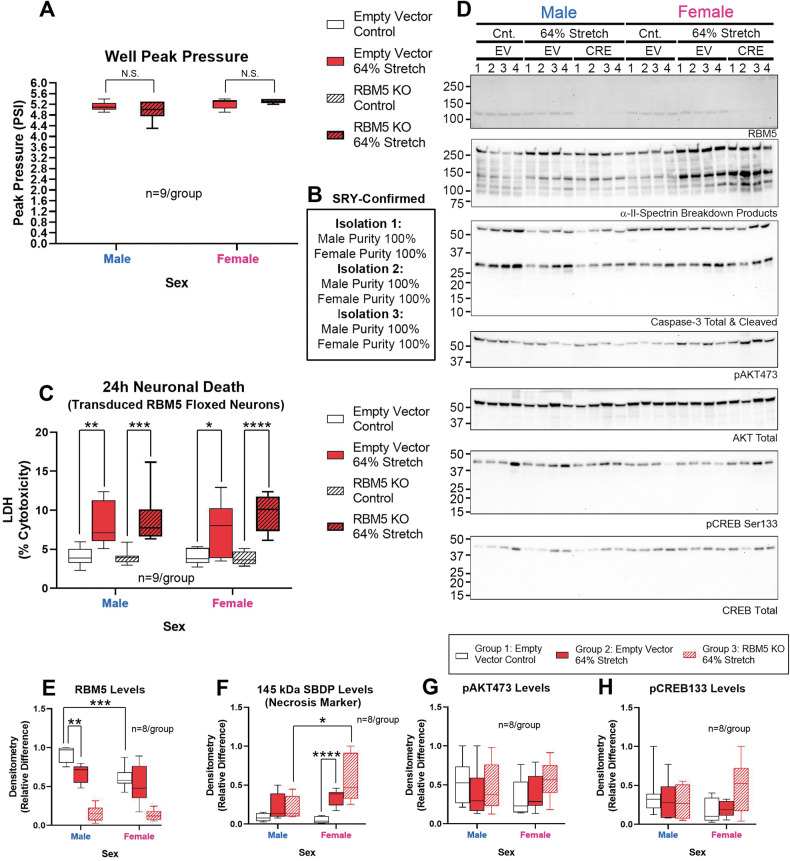
Effect of RBM5 KO and sex on neuronal survival in stretch-injury. **A** Box plots show well peak pressure. **B** Purity of male and female floxed cultures from independent culture isolations in Fig. 4. **C** Box plots show post-injury LDH levels (*n* = 9/group) in control (empty vector) versus CRE (RBM5 KO) neurons. **D** Representative blots (*n* = 4/group) for RBM5, SBDPs, caspase-3, pAKT, total AKT, phosphorylated CREB (pCREB), and total CREB levels. **E**–**H** Densitometric quantification of RBM5, SBDP 145, pAKT and pCREB normalized to total protein stain (*n* = 8/group). Box plots show median, max, min, and IQR. Data were significant at *p* < 0.05. *p < 0.05, **p < 0.01, ***p < 0.001, and ****p < 0.0001. N.S. not significant. RBM5 RNA-binding Motif 5, SBDPs spectrin breakdown products, AKT Protein Kinase B, CREB cAMP response element-binding protein.

Next, we tested if RBM5 KO affects neuronal survival after stretch-injury. Insult severity (~64% stretch) was equivalent in CRE-transduced (RBM5 KO) and EV-transduced (control) floxed neurons across sex (Fig. 4A, B and Table S3). Injury (*p* < 0.0001) but not sex (*p* = 0.9112) or genotype (*p* = 0.2148) affected 24 h post-insult LDH levels (Fig. 4C and Table S3). Germane to cell signaling, endogenous RBM5 levels decreased post-stretch only in male neurons (Figs. 4D, E, S5, S6, S15 and Table S4). SBDPs (145 kDa) were increased in injured female KO neurons vs. injured male KO neurons (Figs. 4D, F, S5, S6 and Table S4). Finally, no post-hoc differences were observed for pAKT or pCREB levels (Figs. 4D, G, H, S5, S6 and Table S4). However, there was a significant interaction (sex x group) for pCREB on the omnibus ANOVA (*p* = 0.0456; Table S4).

**Fig. 5:**
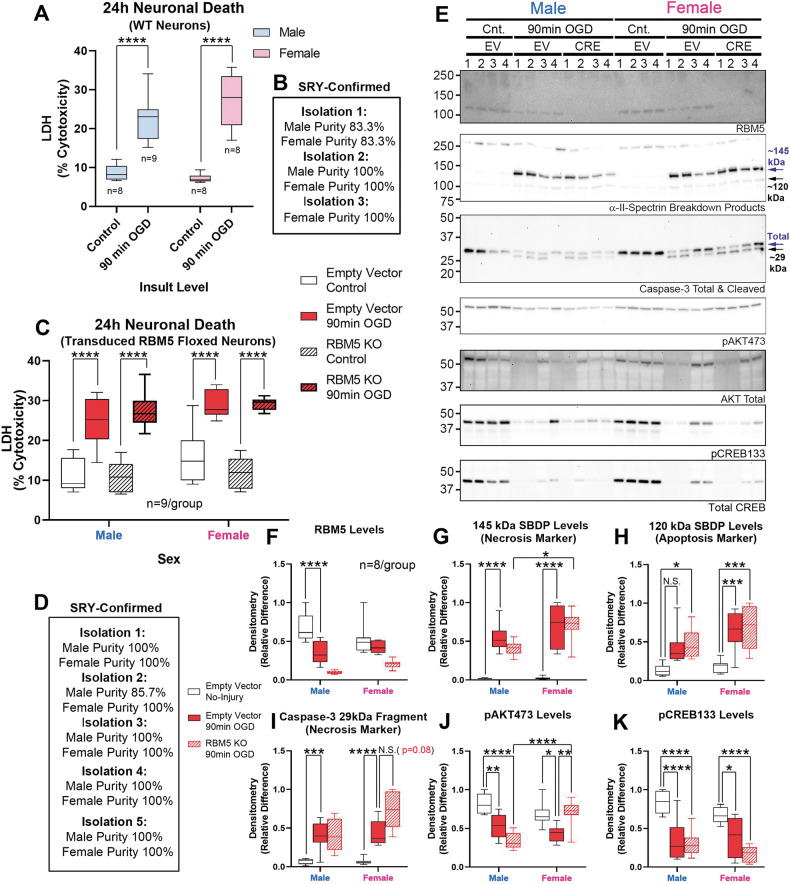
Effect of RBM5 KO and sex on neuronal survival in OGD. **A** Box plots show post-injury LDH levels in sex-separated WT Neurons (*n* = 8-9/group). **B** Purity of WT male and female cultures from independent culture isolations in Fig. 5A. **C** Box plots show post-injury LDH levels (*n* = 9/group) in control (empty vector) versus CRE (RBM5 KO) neurons. **D** Purity of male and female floxed cultures from independent culture isolations in Fig. 5C–K. **E** Representative blots (*n* = 4/group) for RBM5, SBDPs, caspase-3, pAKT, total AKT, pCREB, and total CREB levels. **F**–**K** Densitometric quantification of RBM5, SBDP 145, SBDP 120, caspase-3, pAKT and pCREB normalized to total protein stain (*n* = 8/group). Box plots show median, max, min, and IQR. Data were significant at *p* < 0.05. *p < 0.05, **p < 0.01, ***p < 0.001, and ****p < 0.0001. RBM5 RNA-binding Motif 5, SBDPs spectrin breakdown products, AKT protein kinase B, CREB cAMP response element-binding protein.

Next, we tested if RBM5 KO affects neuronal survival after OGD. No post-hoc sex differences were observed for 24 h LDH levels in male vs. female WT cortical neurons (Fig. 5A, B and Table S5). Injury (*p* < 0.0001) and sex (*p* = 0.0170), but not genotype (*p* = 0.6416) affected 24 h post-insult LDH levels in KO vs. EV-control floxed neurons (Fig. 5C, D and Table S6). Germane to cell signaling, endogenous RBM5 levels were significantly decreased post-injury only in male neurons (Figs. 5E, F, S5, S7, S15 and Table S7). SBDPs (145 kDa) were increased in injured female KO neurons vs. injured male KO neurons (Figs. 5E, 5G, S5, S7 and Table S7). Levels of the SBDP 120 kDa apoptotic marker were increased after OGD but were not affected by RBM5 KO or sex on post hoc testing (Figs. 5E, H, S5, S7 and Table S7). We could not detect the classic apoptotic 17 kDa caspase-3 cleavage product but we did detect the necrotic ~29 kDa caspase-3 fragment produced by calpain cleavage during hypoxia-ischemia [15] (Figs. 5E, I, S5, S7 and Table S7). Pro-survival pAKT activation was significantly decreased after OGD, and this decrease was exacerbated by RBM5 KO in injured male neurons. In contrast, the decrease in pAKT activation was prevented by KO in injured female neurons (Figs. 5E, J, S5, S7 and Table S7). Finally, pCREB levels were decreased after OGD but were not affected by sex or genotype (Figs. 5E, K, S5, S7 and Table S7).

Next, we studied if estrogen treatment alters cell survival in RBM5 KO neurons. ERα protein levels were variable across genotypes, treatments, and injury, and in most cases below the level of detection (Fig. S8). In contrast, G protein-coupled estrogen receptor (GPR30) levels were expressed in both male and female neurons, unaffected by KO, and decreased after OGD (Fig. S9).

**Fig. 6:**
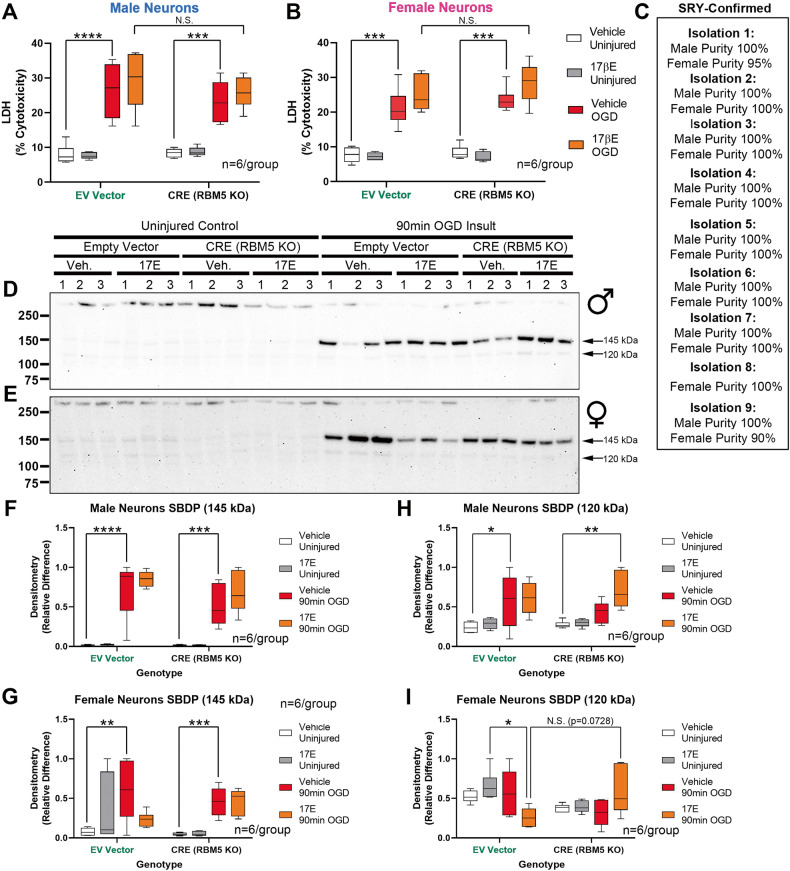
Effect of estradiol on neuronal survival in RBM5 KO neurons in OGD. **A**, **B** Box plots of post-injury LDH levels in male or female neurons treated with or without 1 µM 17β estradiol (E2) (*n* = 6/group). **C** Purity of male and female floxed cultures from independent culture isolations in Fig. 6. **D**, **E** Representative blots (*n* = 3/group) for SBDP levels in male and female neurons, respectively. **F**–**I** Densitometric quantification of SBDP 145 and SBDP 120 normalized to total protein stain (*n* = 6/group). Box plots show median, max, min, and IQR. Data were significant at *p* < 0.05. *p < 0.05, **p < 0.01, ***p < 0.001, and ****p < 0.0001. SBDPs spectrin breakdown products.

Pretreatment with E2 significantly exacerbated 24 h LDH levels only in OGD-injured female neurons, which was detected as a main effect on the omnibus ANOVA (Table S8; *p* = 0.0090) but was not significant for each vector genotype on post-hoc testing (Fig. 6A–C). There was no interaction between genotype and E2 treatment on 24 h LDH levels in male or female neurons (Fig. 6A–C and Table S8). There was also no interaction in male neurons between genotype and E2 treatment on SBDP levels (145 or 120 kDa) (Figs. 6D, F, H, S10A, S11A, and S16 and Table S9). However, in female neurons, while there was no interaction between genotype and E2 treatment on SBDP-145 levels, there was a significant interaction (omnibus ANOVA) on SBDP 120 levels (Figs. 6E, G, I, S10B, and S11B and Table S9), which showed a trend for decreased levels in injured EV-genotype female neurons (Fig. 6I).

### Novel RBM5-regulated protein targets in OGD-injured male and female neurons

**Fig. 7:**
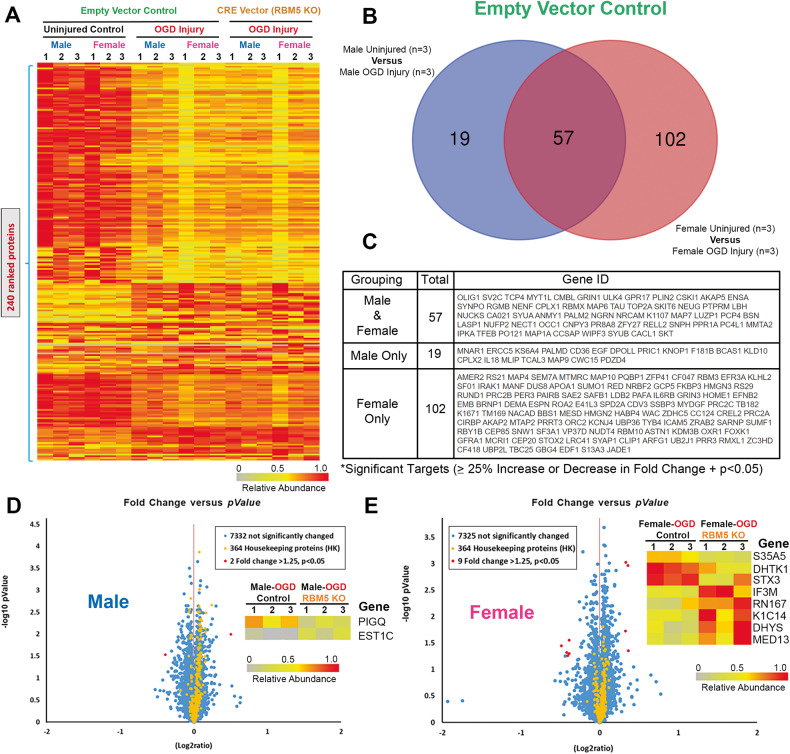
Global proteomic analysis in male and female RBM5 KO neurons in OGD. **A** Dendrogram of 240 differentially expressed (DE) proteins across the 6 treatment groups from three independent culture isolations (*n* = 3/group). **B** Venn diagram on sex differences in protein targets altered 24 h after OGD in EV (control genotype) neurons. **C** Gene IDs of DE proteins after OGD in EV-neurons. **D** Volcano plot of DE proteins in male EV-injured versus male RBM5 KO-injured neurons. **E** Volcano plot of DE proteins in female EV-injured versus female RBM5 KO neurons.

TMT analysis identified 7798 proteins. A total of 240 proteins were differentially expressed (DE) across all manipulations (Fig. 7A and File 1–5). In the control EV-flox neurons, OGD significantly affected the levels of 19 proteins in males only, 102 proteins in females only, and 57 proteins in common in both male and female neurons (Fig. 7B). In the absence of injury, sex had a minor effect on protein levels as only 4 DE targets were identified comparing uninjured-EV male vs. female neurons (Fig. S12A, B and File 6). Excluding RBM5, 2 DE proteins were identified in EV-injured vs. RBM5 KO-injured male neurons, and 8 DE proteins identified in EV-injured vs. RBM5 KO-injured female neurons (Fig. 7D, E, File 7–8, and File 7). LDH analysis confirmed that the insult severity was equivalent in male and female neurons across genotypes (Fig. S13 and Table S10).

## Discussion

We tested if RBM5 inhibition is neuroprotective in isolated neurons and if sex modified the effect of KO on cell survival. A second objective was to elucidate novel proteins regulated by RBM5 in neurons. KO did not affect 24 h survival but increased the necrotic marker SBDP-145 in injured female neurons, consistent with prior work in KOs after a TBI [7]. Hypoxia-ischemia by OGD downregulated the pro-survival kinase AKT (pAKT). RBM5 KO blunted that effect in injured female neurons, whereas loss of pAKT was exacerbated in male KO neurons. Finally, global proteomics revealed major differences in targets regulated by RBM5 in injured male and female neurons.

RBM5 is a potent inducer of apoptosis in cancer cells [2, 3]. In brain, it is abundant in neurons, but little is known about its role(s) in the CNS [5]. RBM5 overexpression in rat cortical neurons increased neuronal death after a mechanical stretch-injury, but subsequent in vivo studies showed no benefit of RBM5 KO on neuronal survival after a moderate-severe CCI [6, 7]. We considered that RBM5 KO in vivo could have unidentified harmful physiological effects that negated any neuroprotective benefit. Contrary to our hypothesis, neuronal RBM5 KO in vitro did not improve 24 h cell survival after traumatic or hypoxic-ischemic insults. Both models primarily induce excitotoxic/necrotic cell death [6, 9–11], and thus RBM5 KO needs to be explored in models inducing neuronal death by other mechanisms such as apoptosis. Furthermore, AKT activation promotes necroptosis in injured neurons [16, 17], SBDP-145 levels are also increased during necroptosis [18], and caspase-2 activity, which is stimulated by RBM5, suppresses necroptosis [19, 20]. Thus, the discontinuity between increased pAKT (anti-apoptotic) and SBDP-145 levels (necrosis marker) in injured female KO neurons raise the possibility that RBM5 could be a novel regulator of necroptosis.

The effect of RBM5 in injured neurons is sex-dependent. Post-insult SBDP and pAKT473 levels were markedly changed, and the results of the global proteomic analysis were fundamentally different, in male versus female KO neurons. Injured male KO neurons had decreased levels of *Phosphatidylinositol N-Acetylglucosaminyltransferase Subunit Q* (PIGQ) and increased levels of *Carboxylesterase 1C* (EST1C). This may be harmful given that PIGQ is essential for glycosylphosphatidylinositol (GPI)-anchor biosynthesis and thus critical for numerous signaling pathways [21]. Indeed, impaired PIGQ variants result in infantile epileptic encephalopathy [22]. Also, EST1C is a serine hydrolase that can promote inflammation [23].

RBM5 KO in injured female neurons decreased levels of *Solute Carrier Family 35 Member A5* (SLC35A5), *Probable 2-Oxoglutarate Dehydrogenase E1 Component DHKTD1, Mitochondrial* (DHTK1), and *Syntaxin 3* (STX3), and conversely increased levels of *Mitochondrial Translational Initiation Factor 3* (IF3M), *Ring Finger Protein 167* (RN167), *Keratin-14* (K1C14), *Deoxyhypusine Synthase* (DHYS), and *Mediator of RNA polymerase II transcription subunit 13* (MED13). Impaired nucleoside-sugar transporter SLC35A5 is associated with neurological dysfunction [24, 25]. Mutations in the mitochondrial regulator DHTK1 is associated with amyotrophic lateral sclerosis [26–29]. STX3 modulates axonal trafficking and exocytosis [30, 31], and we and others reported a link between RBM5 and endocytosis [14, 32]. Dysfunction of the mitochondrial protein synthesis regulator IF3M is associated with Parkinson’s disease [33]. RN167 is a E3 ubiquitin ligase that regulates surface expression of neuronal AMPA receptors [34, 35]; linking RBM5 with the regulation of a major neurotransmitter system. K1C14 is an intermediate filament normally found in keratinocytes but temporarily expressed during neuronal development [36]. DHYS is an enzyme critical for the synthesis of hypusine (a unique amino acid) [37]; deletion of DHYS in mice caused severe learning and memory impairment [38]. Finally, MED13 is part of a complex that regulates transcription initiation and MED13 mutations are linked with neurodevelopmental and neurodegenerative disorders [39, 40].

Relevant to our findings, evidence suggests that RBM5 activity varies in the human population. A GWAS on 188,578 individuals found that a small nucleotide polymorphism in an RBM5 gene intron (rs2013208; MAF = 0.5) is associated with high HDL levels and coronary artery disease [41]. Furthermore, a follow-up study in Asians found that rs2013208 is associated with sex differences in serum lipid profiles [42].

Because ERα is increased in the hippocampus in female RBM5 KOs with TBI, we explored if E2 might affect neuronal survival in KO neurons [7]. E2 did not alter the effect of KO on neuronal survival, and we could not replicate the finding of increased ERα protein levels in female KO neurons. That discrepancy might result from using cortical neurons here, or because increased ERα protein in whole hippocampal KO extracts was derived from non-neuronal cell types underrepresented in our culture system [43].

E2 pretreatment exacerbated cell death only in female neurons. Whether that effect results from the use of a next-generation media merits study. Next-generation media increases the abundance of proteins that modulate electrophysiology, and increases the ratio of GABAergic to glutamatergic neurotransmission in a manner mimicking the brain [6, 12]. This could explain our observations in female neurons because GABAergic signaling is excitatory in neonates, promotes neuronal death, and E2 treatment can exacerbate that effect [44]. Indeed, E2-mediated augmentation of GABA receptor-mediated calcium influx is manipulatable at least until DIV7 in primary neurons in vitro, and remains unexplored in next-generation media [45]. Also, while ERα levels were low, GPR30 was ubiquitously expressed, and communicates some of the effects of E2 on GABAergic signaling [46].

Additional strengths of our study included implementing a rigorous protocol to investigate sex in primary cultures. Over 450 embryos were genotyped to calculate the purity of male versus female cultures. We achieved a ~97% success rate to correctly assign embryo sex via necropsy but not all attempts were successful, and we set thresholds to protocolize culture exclusions. We also established protocols to optimize the study of cell signaling in neuronal stretch. Finally, we performed global proteomic analysis in OGD-injured pure male or female cells maintained in next-generation Neurobasal-Plus media, producing interesting sex differences.

There were also limitations. A single post-injury time point (24 h) and single insult level were assessed in both models. The 64% stretch and 90 min OGD were selected because they represent an intermediate insult level. We cannot exclude the possibility that RBM5 KO may have different effects at other insult severities. LDH release was the primary cell death outcome. LDH is a low-bias and high-sensitivity test that mainly detects early necrosis and late apoptosis, and may not capture all RBM5-modulated aspects of cell death [47]. We did not assess the impact of RBM5 on neuronal death induced by other insults triggering different cell-death mechanisms such as apoptosis or other specific programmed necrosis pathways. Also, RBM5 contains 6 major functional domains that target different sets of genes [1]. The RRM1/2 domains mediate cell death in cancer cells [4]. Thus, a tailored approach to RBM5 inhibition may unmask neuroprotection in the excitotoxicity models used here. EV-floxed neurons were used as controls (i.e., functionally equivalent to WT neurons). It was impractical to repeat studies in sex-separated WT neurons given the scope of experiments. However, our findings agreed with in vivo studies in TBI which included WT mice for comparison [7]. We did not perform an extensive comparison with last generation culture media, which is still frequently used. Lastly, follow-up studies are needed to independently confirm the results of the global proteomic analysis.

In summary, in vitro inhibition of the tumor suppressor gene RBM5 did not decrease cell death in cortical neurons after a mechanical or hypoxic-ischemic insult. However, RBM5 modulates cell signaling after neuronal injury in a sex-dependent manner. Our findings support a growing body of evidence that, in brain, RBM5 preferentially modulates proteins linked with brain function, unlike its role in cancer [7, 32, 48].

## Materials and methods

### Reagents

*PVDF Membrane*: 0.2 µM pore size, Cat# SVFX8301XXXX101 (MDI Technologies; Harrisburgh, PA, USA). Gibco B-27 Plus Neuronal Culture System: Neurobasal Plus with B27 Plus Supplement Cat#A3653401 (ThermoFisher). *Neuronal Culture Media*: Gibco B-27 Plus Neuronal Culture System: Neurobasal Plus with B27 Plus Supplement Cat#A3653401 (ThermoFisher). *Cell culture reagents*: L-glutamine, Cat#25030081 (ThermoFisher). Penicillin/Streptomycin Cat#15140-122 (Fisher). Glutamate, Cat#G8415 (Sigma). HBSS, Cat#14175103 (ThermoFisher). HEPES, Cat#15630080 (ThermoFisher). NaHCO_3_, Cat#G8415 (Sigma). Trypsin, Cat#LS004425 (Worthington Biochemical). Fetal bovine serum, Cat#26140079 (ThermoFisher). HEK 293 T cells, Cat#LT008, Lot#G12CL67D (GeneCopoeia), Opti-MEM, Cat# Cat#31985-070 (Gibco), Matrigel® Basement Membrane Matrix Phenol Red Free, Cat#356237, Lot#0069011, Lot#102001, Lot#1069003 (Corning). *Drugs*: 17β-estradiol, Cat#E2758, Lot#SLCN3888 (Sigma). *Plasmids and Lentivirus Kits*: GeneCoepeia Lentivirus Packaging Kit, Cat#LT002 (GeneCopoeia), One Shot^TM^ TOP10 Chemically Competent *E. coli*, Cat#C404010 (ThermoFisher), Macherey-Nagel NucleoBond Xtra Midi Plus EF Midi kit for endotoxin-free plasmid DNA, Cat#NC1774747 (ThermoFisher) QuickTiter Kit, Cat# VPK-107 (Cell Biolabs, Inc). *Cell Injury Supplies:* LDH Release Assay Kit, Cat#ab65393 (Abcam), BioFlex untreated culture plates, Cat#BF-3001U (FlexCell International). Cell Recovery Solution, Cat#354253, Lot#0281003 (Corning). *Primary Antibodies*: α-Fodrin, Cat#BML-FG6090, Lot#08231207 (Enzo); pan-AKT, Cat#4691, Lot#28 (Cell Signaling Technologies); pan-AKT, Cat#2920 S, Lot#3 and #8 (Cell Signaling Technologies); phosho-AKT (Ser473), Cat#4060 S, Lot#23 and #27 (Cell Signaling Technologies); ERK, Cat#4696 S, Lot#22 (Cell Signaling Technologies); phospho-ERK, Cat#4377 S, Lot#10; PERK, Cat#LS-C757915, Lot#143906 (LSBio); RBM5, Cat#19930-1-AP, Lot#00049598 (ProteinTech); Caspase-3, Cat#19677-1-AP Lot#00094596 and #00109139; GPR30, Cat#PA5-28647, Lot#YD3884109C (ThermoFisher); Estrogen Receptor α, Cat#ab76228, Lot#1016753-3 (Abcam). *Secondary antibodies*: Goat anti-Rabbit HRP, Cat#G-21234 (ThermoFisher), Goat anti-Mouse, Cat#G-21040 (ThermoFisher).

### Animals

Animal work was approved by the IACUC of the University of South Florida (USF). Methods of euthanasia adhered to the AVMA Guidelines for the Euthanasia of Animals. The development of RBM5^tm1Ozg^ mice on a C57BL/6 background was described previously [48]. Conditional floxed mice express RBM5 normally until mated with mice that express the CRE recombinase protein, or until primary cells derived from homozygous conditional floxed mice are transduced in vitro with a viral vector to express CRE. Founders were provided to the USF by the University of Pittsburgh upon execution of an MTA. Adult dams were maintained on a 12 h light/dark cycle and had ad libitum access to food and water.

### Genotyping

Mouse genotyping was described previously [48]. Mice were bred in house. The primer pair used to differentiate wild-types (WT) from mice harboring the floxed allele is: **F-**GCGGGACTCAGATTACAAAAGATC and **R-**TGCAGCCTATCTTCTATAAGGG. The WT allele yields a 317 bp PCR product and the floxed allele a 420 bp product. Embryonic tail snips were collected at the time of sex stratification and stored in 70% ethanol in PCR tubes at 4 °C. The primer pair used to differentiate males from females is: **F-**TTGTCTAGAGAGCATGGAGGGCCATGTCAA and **R-**CCACTCCTCTGTGACACTTTAGCCCTCCGA. The SRY PCR product is 273 bp. Gel images of SRY genotyping for all embryos are available in the supplementary (Table S11 and File 9).

### Sex dichotomized primary neuronal culture

Timed-pregnant mice were euthanized between E17-19 by rapid CO_2_ euthanasia followed by cervical disruption, and embryos quickly euthanized by cervical disruption. Brain cortices were harvested in ice cold dissection media (HBSS, 15 mM HEPES, 10 mM NaHCO_3_, 1X PEN/STREP, pH 7.6). Each embryo was given a unique numerical ID, and the brain/body were separated into two 35 mm dishes. The process was repeated for all embryos per litter. Sex organs were identified in the abdominal cavity under a dissecting microscope and all male or female cortices were combined for downstream tissue dissociation. Tail snips were collected from all embryos for later SRY genotyping, which permitted retrospective quantification of the true % purity for “male” versus “female” neurons across cultures. A threshold of ≥80% sex-specific cell purity was protocolized for study inclusion. The order in which pooled cortices (male or female) were processed was alternated across culture isolations to avoid introducing bias by potential slight differences in the starting baseline viability. Sex-sorting procedures were omitted for standard “mixed-sex” cultures. Cortices were mechanically dissociated with scissors for 2 min in dissection media, trypsinized 8 min in 37 °C water bath (HBSS, 1 mg/mL trypsin, 4 mg/mL DNaseI, pH 7.6), and triturated with a fire-polished glass pipet (~10 passes in Neurobasal Plus supplemented with 10 mg/mL DNaseI). Cell counts and baseline viability were measured using a Countess Automated Cell Counter (ThermoFisher). Cells for stretch-injury studies were seeded onto Matrigel (0.5 mg per well) pre-coated six-well Silastic plates at a density of 1.5 × 10^6^ neurons per well. Cells for OGD were seeded onto poly-D-lysine (0.05 mg/mL) coated 35-mm dishes at a density of 2.3 × 10^6^ per dish. Homozygous RBM5 floxed cells (expressing RBM5 protein at normal levels) were treated with either an empty control vector or a CRE-recombinase overexpressing vector (i.e., to induce RBM5 KO). A multiplicity of infection (MOI) of 100 MOI was chosen based on preliminary MOI dose-response studies in cortical neurons (Fig. S17). A power analysis was not done due to the lack of preexisting data on the effect that Neurobasal-Plus next-generation media has on injury outcomes in the models used here, and due to a lack of data obtained from cells isolated from transgenic conditional RBM5 KO mice. Sample size was estimated based on our prior experience in stretch-injury studies in primary rat and mouse neurons [6, 49]. The number of independent culture isolations performed for each experiment is provided in the figure legends and/or figures. Randomization and blinding of experimental groups in vitro were not performed.

### Plasmid amplification and high-titer lentivirus production

The pReceiverLv125 expression plasmid and packaging plasmid system were used (GeneCopoeia). Plasmids (5 ng expression and packaging) were transformed in 50 µL of competent *E. coli*, heat shocked at 42 °C, and incubated in 250 µL of 37 °C S.O.C. broth for 1 h at 225 rpm. Colony selection was performed on LB Agar plates containing 100 mg/L of ampicillin, and expanded in LB broth with 100 mg/L ampicillin (37 °C for 8–12 h at 225 rpm). Bacteria were harvested by centrifugation (6000 × *g* for 10 min), and plasmid DNA isolated by MidiPrep following the manufacture instructions. Plasmid concentrations and purity were measured on a Nanodrop. HEK293T cells were expanded to ~80–90% confluency in Opti-MEM/10%FBS/1X pen/strep (Complete Growth Media, CGM) on poly-d-lysine -coated T-225 flasks. Expression, packaging plasmids, and EndoFectin were added to T-225 flasks in 13 mL of CGM. At ~12 h post-transfection, medium was replaced with 18 mL of fresh CGM and supplemented with TiterBoost (1:500). The viral soup was harvested at 33 h post-transfection and again at 50 h post-transfection. Collections originating from the same flasks were centrifuged at 2000rpm/4 °C/5 min to remove debris, combined, filtered via a 150 mL 0.45 µM PES filter top system, concentrated via a Centricon-Plus 70 filtration unit (2500 rpm/4 °C/50 min), and ultracentrifuged using a TI-32 Swing-Bucket rotor (Beckman) for 1.5 h at 24,000 rpm/4 °C in a 30 mL conical centrifuge tube pre-loaded with 2 mL of sterile 20% sucrose in PBS. The supernatant was removed, and the pellet covered in 200 µL of sterile cold Opti-MEM overnight at 4 °C. The viral pellet was mixed via gentle pipetting and 15–20 µL aliquots stored at −80 °C. Aliquots were only used once to avoid decreased viral transduction efficiency from multiple freeze-thawing. Viral titer was assessed using the Lentivirus-Associated HIV p24 ELISA QuickTiter Kit (Cell BioLabs) following the manufacture instructions.

### Mechanical stretch injury

Mechanical stretch-injury of cultured neurons was done as described [6]. The achieved well peak pressure corresponds with the % biaxial deformation of the Silastic membrane (% stretch). In brief, neurons on Matrigel-coated BioFlex plates were injured at DIV10 by a 100% nitrogen gas burst delivered in 75 ms via the Cell Injury Controller (CIC) II (Custom Design & Fabrication Inc.; Sandston, VA, USA). The media/cells were harvested 24 h later (DIV11) for endpoint analysis. Immediately prior to injury, well volumes were adjusted to 1 mL via a complete media exchange. For gene KO and drug treatment stretch-injury studies, Matrigel removal was omitted to preserve protein phosphorylation, and protocolized across groups.

### Oxygen-glucose deprivation

Sterile balanced salt solution (BSS) was prepared in ddH_2_O (116 mM NaCl, 5.4 mM KCl, 1.8 mM CaCl_2_*2H_2_O, 0.8 mM MgSO_4_*7H_2_O, 26.2 mM NaHCO_3_, 1.0 mM Na_2_HPO_4_, 0.01 mM glycine, pH 7.4). In preparation for injury, BSS was degassed in 95% N_2_/5% CO_2_ at 37 °C. Control neurons received identical manipulations, but the BSS was supplemented with 500 mM glucose (D-glucose) and were normoxic. The glucose concentration in BSS controls was the same as in Neurobasal-Plus, which was confirmed telephonically by a ThermoFisher Technical Specialist. The remaining components/concentrations of ingredients in next-generation Neurobasal-Plus media are proprietary. At DIV9, 1 mL of conditioned media was collected and saved from each 35 mm dish and pooled separately by group (e.g., virus/genotype and/or sex). “Resuscitation Media” (RM) was prepared by mixing (1:1) conditioned media with fresh Neurobasal/B27-Plus. Remaining media was then aspirated off, cells washed once with 2 mL warm BSS, then replaced with 2 mL of 37 °C deoxygenated BSS, or with oxygenated BSS+glucose for controls. Neurons subjected to injury were placed in a hypoxia chamber (MIC-101, Billups-Rothenberg, Inc.) flushed with 95% N_2_/5% CO_2_ for 5 min at a flow rate of 20 L/min, and sealed in an anoxic environment. The chamber was transferred to a standard 37 °C incubator for 90 mins. Control neurons were placed directly in the incubator to maintain normoxia. The BSS was removed and replaced with 1 mL of RM and neurons returned to the incubator for an additional 24 h recovery period.

### Estrogen studies

A 250 μM stock solution of E2 was prepared in 100% ethanol, syringe filtered, aliquoted, and stored at −30 °C. Controls received an equal concentration of sterile ethanol without E2. For OGD/estrogen studies, 1 μM E2 was added to the growth media DIV6-9 (72 h pretreatment), followed by addition to the RM media (at 1 μM) for the 24 h post-injury period (4d total exposure). The 1 μM E2 concentration was chosen based on reports of neuroprotection in cortical and hippocampal neurons at that dose [50, 51]. The pre- and post-treatment design was to model the in vivo scenario in which circulating estrogen would be available to target neurons in the brain before and after a CNS injury in a non-ovariectomized female rodent. Media and protein extracts were harvested at DIV10 for LDH-release and for western blot analysis, respectively. We confirmed telephonically with a ThermoFisher Technical Specialist that Neurobasal-Plus and B27-Plus does not contain estrogen or testosterone.

### LDH assay

Media was collected from wells or dishes at 24 h post-injury. Samples were stored at −80 °C. For each 6-well stretch-plate, and for OGD experiments involving 35-mm dishes, one uninjured well or dish per group were briefly incubated (15 min at 37 °C) with cell lysis buffer on the day of media harvesting (Abcam, 100 μL per 1 mL of media). This provided maximum LDH values across groups (e.g., for each sex and/or genotype) to calculate % cytotoxicity [= (sample value − negative control) / (maximum LDH control − negative control) × 100]. Media was added to 96-well plates and LDH levels were measured using manufacture protocols. Absorbance (OD450) was recorded on a Accuris™ SmartReader™ 96 well plate reader (Accuris™).

### Western blot

Cultured neurons were washed in 1X PBS then harvested in RIPA buffer with EDTA, protease inhibitors and phosphatase inhibitors (65-80 μL), briefly sonicated, and centrifuged (10 min, 15,000 × *g*). Supernatant was transferred to fresh tubes and stored at −80 °C. Protein concentration was calculated using the BCA method (ThermoFisher Scientific). Protein was loaded onto 26-well Criterion TGX gels at 20 μg/well and electrophoresed at 200 V in a Criterion Cell (BioRad). Protein was transferred to a PVDF membrane via the Criterion Blotter plate-electrode system (BioRad) (40 min/100 V/4 °C). Membranes were stained Swift Membrane Stain™ (Fisher Scientific), and the results scanned at 600dpi for densitometry on total protein loading. All membrane stains are available in the supplementary. Blots were blocked in 7.5% milk in TBST for 1 hr. Primary (overnight, 4 °C) and secondary (2 h, RT, 1:15,000) antibodies were incubated in 7.5% milk in TBST with TBS rinses (3X, 5 min, RT). Membranes were developed with ECL-2 detection substrate (ThermoFisher Scientific) and imaged on 9.1MP iBright CL1500 Imaging System ThermoFisher Scientific). In many cases the membranes were cut into halves immediately post-transfer to permit simultaneous investigation of 2 targets (i.e., due to low sample availability). Additional digital trimming was done in photoshop to organize data into primary figures, but the original uncropped full or sectioned membranes are available in the supplementary.

### TMT analysis

Each sample (*n* = 1) for TMT was generated by combining neurons from two 35 mm dishes with identical variables (i.e., pooling *n* = 2 biological replicates). Each culture isolation yielded 12 dishes and after combining pairs of dishes by group, a total of 6 samples (*n* = 1 per group for sex, viral manipulation, and injury). This was repeated for 3 independent culture isolations, giving a final yield of 18 samples (cell pellets). Samples were flash frozen and shipped on dry ice to Poochon Scientific (*n* = 3 group). Vials were coded and Poochon staff performing TMT were blinded to groups. Protein concentrations were measured using the BCA method. 100 µg of total protein per sample was processed for MS analysis. In brief, samples were trypsinized, peptides subjected to isobaric labeling using a TMT-18plex kit (ThermoFisher), dried in a Thermo Savant ISS110 vacuum concentrator, and kept at -80°C. Peptides were resuspended in 10 mM TEABC and labeling efficiency was verified to be ≥95%. Fractionation was done on an Agilent AdvanceBio Column (2.7 μm, 2.1 × 250 mm) and with an Agilent UHPLC 1290 system. Separation was achieved via a gradient of Solvent A (10 mM TEABC, pH 8.0) and Solvent B (10 mM TEABC, pH 8.0, 90% ACN) at 250 µL/min. Fractions were collected on a 96-well plate using a 1260 series auto-sample fraction collector. The 96 fractions were combined into 24 fractions according to collection time for LC/MS/MS analysis. LC/MS/MS was done on a Thermo Scientific Orbitrap Exploris 240 Mass Spectrometer and a Thermo Dionex UltiMate 3000 RSLCnano System. Each peptide fraction (24 total) was loaded onto a peptide trap cartridge at a flow rate of 5 µL/min and eluted onto a reversed-phase 25 cm C18 EasySpray nano column (Thermo) using a linear gradient of acetonitrile (3–36%) in 0.1% formic acid. Eluted peptides from the EasySpray column were ionized and sprayed into the mass spectrometer, using a EasySpray Ion Source (Thermo; spray voltage, 1.6 kV, capillary temperature, 275 °C). The 24 fractions were sequentially analyzed. The Exploris 240 instrument was operated in the data dependent mode to automatically switch between full scan MS and MS/MS acquisition. Survey full scan MS spectra (m/z 350 − 1800) was acquired in the Orbitrap with 35,000 resolutions (m/z 200) after an accumulation of ions to a 3 × 106 target value based on predictive automatic gain control (AGC). The maxima injection time was set to 100 ms. The 20 most intense multiply charged ions (*z* ≥ 2) were sequentially isolated and fragmented in the octopole collision cell by higher-energy collisional dissociation (HCD) using normalized HCD collision energy 30 with an AGC target 1 × 105 and a maxima injection time of 400 ms at 17,500 resolutions. The isolation window was set to 2 and fixed first mass was 120 m/z. The dynamic exclusion was set to 20 s. Charge state screening was enabled to reject unassigned and 1+, 7+, 8+, and >8+ ions. Raw data files acquired from analysis of the 24 fractions were searched against mouse protein sequences database obtained from UniprotKB website using the Proteome Discoverer 2.4 software (Thermo, San Jose, CA) and based on the SEQUEST and percolator algorithms. The false positive discovery rate (FDR) was set to 1%. TMT-tag based quantification was used for determining the relative abundance of proteins identified in the 18 samples. Relative protein abundance was calculated as the ratio of abundance determined by the TMT-tags. A normalization of the ratio (relative abundance) was performed by using the summed reporter ion intensities. Gene Ontology (GO), Kyoto Encyclopedia of Genes and Genomes (KEGG) annotation, Reactome Pathway Database annotation and GO molecular function and biological process categories were obtained from UniprotKB protein database online tool.

### Statistics

Western blot densitometry of total protein stain (TPS) and individual targets were measured with UN-SCAN-IT software (Silk Scientific). For each blot, we divided TPS normalized densitometric values by the largest normalized intrablot value to distribute the results on a 0–1 scale to express the data as the “relative difference” for between group comparisons, as we have reported [52]. Data on individual targets from multiple blots were combined and normality assessed using the Kolmogorov-Smirnov test. If passed, a standard parametric factorial ANOVA or 2- or 3-Way-ANOVA was used. If failed, a Kruskal-Wallis was applied for factorial ANOVA. For non-parametric multifactorial data, the data were transformed using the Aligned Rank Transformation (ART) tool [53]. The ranked data were then analyzed by standard factorial ANOVA for each individual factor (i.e., genotype, injury, interaction term) or unpaired t-test to obtain omnibus p-values. For pairwise comparisons, a fourth ranking was done using the ART-Contrasts (ART-C) algorithm [54], which does not inflate Type I error rates on post-hoc testing. Dunnett’s or Tukey’s multiple comparison was used for post-hoc significance tests in parametric ANOVAs. Dunn’s multiple comparison was used for post-hoc significance tests with non-parametric Kruskal–Wallis ANOVA. Data were significant at *p* < 0.05 and graphed using GraphPad Prism. Box plots show median, max, min, and IQR. *TMT Studies:* Group comparisons were assessed using a Student’s *t*-test. Proteins were considered differentially expressed between groups if (a) the ± fold change was ≥25% and (b) the *p* < 0.05. The average, standard deviation, coefficient of variation, and paired T-test (*p*-values) were calculated in Microsoft Excel. All tests were two-sided.

### Supplementary information

Data Set 1

Data Set 2

Data Set 3

Data Set 4

Data Set 5

Data Set 6

Data Set 7

Data Set 8

Data Set 9

Supplementary Material

## Data Availability

All data generated or analyzed during this study are included in this published article and its supplementary information files.
