# Supplementary material for: Neuronal RBM5 modulates cell signaling responses to traumatic and hypoxic-ischemic injury in a sex-dependent manner
Source: Cell Death Discov. 2023 Oct 17;9:379. doi: 10.1038/s41420-023-01677-7 (PMC10582027; doi:10.1038/s41420-023-01677-7)
Supplement: Supplementary file 10 — Supplementary Material [file 41420_2023_1677_MOESM10_ESM.pdf]

Supplementary Figure Legends

Fig. S1: Total Protein Stains and Additional Blot for SBDP Following Mechanical Stretch Injury in Mixed-Sex WT Neurons. (A) Total protein stain of the PVDF membrane for densitometry as a loading control for sample set 1-3 on SBDPs (i.e., Fig. 1C). **(B)** Western shows SBDPs (n=3/group) for sample set 4-6. **(C)** Total protein stain for densitometry as a loading control for sample set 4-6 on SBDPs (Fig. S1B).

Fig. S2: **Additional Western Blots for Signaling Targets in Fig. 2. (A-F)** Western blots show target analysis results for sample set B on pERK, ERK total, pAKT, AKT Total, PERK, and GFAP (n=2/group). Results of sample set A and set B were combined to generate box plots in Fig. 2.

Fig. S3: Total Protein Stains for Western blots on Cell Signaling Targets in Fig. 2 Studies. (A-I) Total protein stains of PVDF membranes used for densitometry as a loading control in Fig. 2 and Fig. S2.

Fig. S4: Total Protein Stains and Additional SBDP Blot in Sex-Separated WT Neurons Subjected to Mechanical Stretch Injury. (A) Total protein stain of the PVDF membrane for densitometry as a loading control for sample set 1-4 on SBDPs (i.e., Fig. 3D). **(B)** Western shows SBDPs (n=4/group) for sample set 5-8. **(C)** Total protein stain for densitometry as a loading control for sample set 5-8 on SBDPs (Fig. S4B).

Fig. S5: Additional Western Blots for Signaling Targets in Fig. 4 and Fig. 5 on the Effect of RBM5 KO in Mechanical Stretch and OGD Injuries. (A) Western blots show the results of RBM5, SBDP, caspase-3, pAKt, AKT total, pCREB, and CREB total for sample set 5-8 (n=4/group) in the stretch injury model. Data were combined with results of sample set 1-4 in to generate graphs in Fig. 4. **(B)** Western blots show the results of RBM5, SBDP, caspase-3, pAKt,

AKT total, pCREB, and CREB total for sample set 5-8 (n=4/group) in the OGD model. Data were combined with results of sample set 1-4 to generate graphs in Fig. 5. The red asterisk indicates a single sample for caspse-3 in the stretch-injury group for which analysis was not possible due the lack of sample availability.

Fig. S6: Total Protein Stains for Cell Signaling Targets Measured in Fig. 4 and Fig. S5A in Stretch-Injury. (A-F) Total protein stain of PVDF membranes for densitometry as a protein loading control. Membranes correspond with cell signaling targets shown in Fig. 4D and in Fig. S5A.

Fig. S7: Total Protein Stains for Cell Signaling Targets Measured in Fig. 5 and Fig. S5B in OGD. (A-F) Total protein stain of PVDF membranes for densitometry as a protein loading control. Membranes correspond with cell signaling targets shown in Fig. 5E and in Fig. S5B.

Fig. S8: ERα Protein Levels in Sex-Separated EV-Control Versus RBM5 KO Neurons in the OGD Model. EV-control or CRE (RBM5 KO) neurons were pre-treated 3d with 1μM 17E or vehicle, injured with a 90min OGD, and returned to the incubator for an additional 24h with 1μM 17E or vehicle. **(A-B)** Western Blots for ERα and the corresponding total protein stains of PVDF membranes in male neurons. Panel A shows sample set 1-3, and panel B shows sample set 4-6. Total protein stain was collected after protein transfer and prior to incubation with a primary antibody. **(C-D)** Western Blots for ERα and the corresponding total protein stains of PVDF membranes in female neurons. Panel C shows sample set 1-3, and panel D shows sample set 4-6.

Fig. S9: GPR30 Protein Levels in Sex-Separated EV-Control Versus RBM5 KO Neurons and in the OGD Model. (A) Western blot for GPR30 and the corresponding total protein stain in

uninjured male and female neurons of the control (EV) and KO (CRE) genotype. Sample set A (1-3) and set B (4-6) were run together on the same blot. No effect of KO was seen. **(B and C)** Western Blots for GPR30 and the corresponding total protein stains of PVDF membranes in male neurons in EV vs. KO, vehicle vs. 17E, uninjured and OGD-injured neurons. Panel B shows sample set 1-3, and panel C shows sample set 4-6. No effect KO or 17E was seen, but there was a robust downregulation of GPR30 at 24h post-injury.

Fig. S10: Total Protein Stains for Sample Set 1-3 on SBDP Blots in Fig. 6. (A) Total Protein stain of PVDF membrane corresponding to sample set 1-3 Western Blot on SBDPs in male neurons (Fig. 6D). **(B)** Total Protein stain of PVDF membrane corresponding to sample set 1-3 Western Blot on SBDPs in female neurons (Fig. 6E).

Fig. S11: Additional Blots on SBDP for Sample Set 4-6 in Support of Fig. 6 Studies. (A) Western blot on SBDPs in sample set 4-6 and the corresponding total protein stain of PVDF membrane for densitometry as a loading control in male neurons. **(B)** Western blot on SBDPs in sample set 4-6 and the corresponding total protein stain of PVDF membrane for densitometry as a loading control in female neurons.

Fig. S12: Volcano Plot on Differentially Expressed Genes Comparing Male vs. Female Uninjured EV-Control Neurons. (A) Volcano plot shows the fold-change and p-value of the proteins identified in TMT analysis comparing uninjured EV male neurons versus uninjured EV female neurons. **(B)** Heat map of the 4 DE proteins.

Fig. S13: Culture Purity (Sex Dichotomization) and Insult Severity in Cultures for TMT Analysis. (A) All 3 independent floxed cortical neuron cultures for TMT Analysis were confirmed by SRY genotyping of embryos to be 100% male or 100% female. **(B)** Box plots show 24h post-

injury raw LDH values (OD450). There were no differences in insult severity comparing male vs. female EV injured neurons or male vs. female KO injured neurons.

Fig. S14: Uncropped Blots from Fig. 2. Images show the complete set of uncropped western blots to support Fig 2 studies.

Fig. S15: Uncropped Blots from Fig. 4 and Fig. 5. Images show the complete set of uncropped western blots used to support Fig. 4 and Fig. 5 studies. Some blots were physically cut in half after transfer to the PVDF membrane to permit the analysis of multiple targets at one time. This was necessary to conserve sample, which can only be obtained in limited quantities from 6-well plates.

Fig. S16: Uncropped Blots from Fig. 6. Images show the complete set of uncropped western blots to support Fig 6 studies.

Fig. S17: Validation of Lentivirus Dose (Multiplicity of Infection) in Floxed Neurons for KO Studies. **(Top)** Western blot shows RBM5 expression DIV6 cortical neurons in EV transduced vs. CRE transduced floxed neurons at 30, 60, and 100 MOI (n=3/group). **(Middle)** Western blot shows CRE Recombinase expression in EV transduced vs. CRE transduced floxed neurons at 30, 60, and 100 MOI (n=3/group). **(Bottom)** Representative total protein stain. 100 MOI was selected as the optimal dose due to maximal inhibition of RBM5 expression and confirmation of CRE expression. RBM5 expression was not inhibited by the EV control vector.

Supplementary Figures

Fig. S1

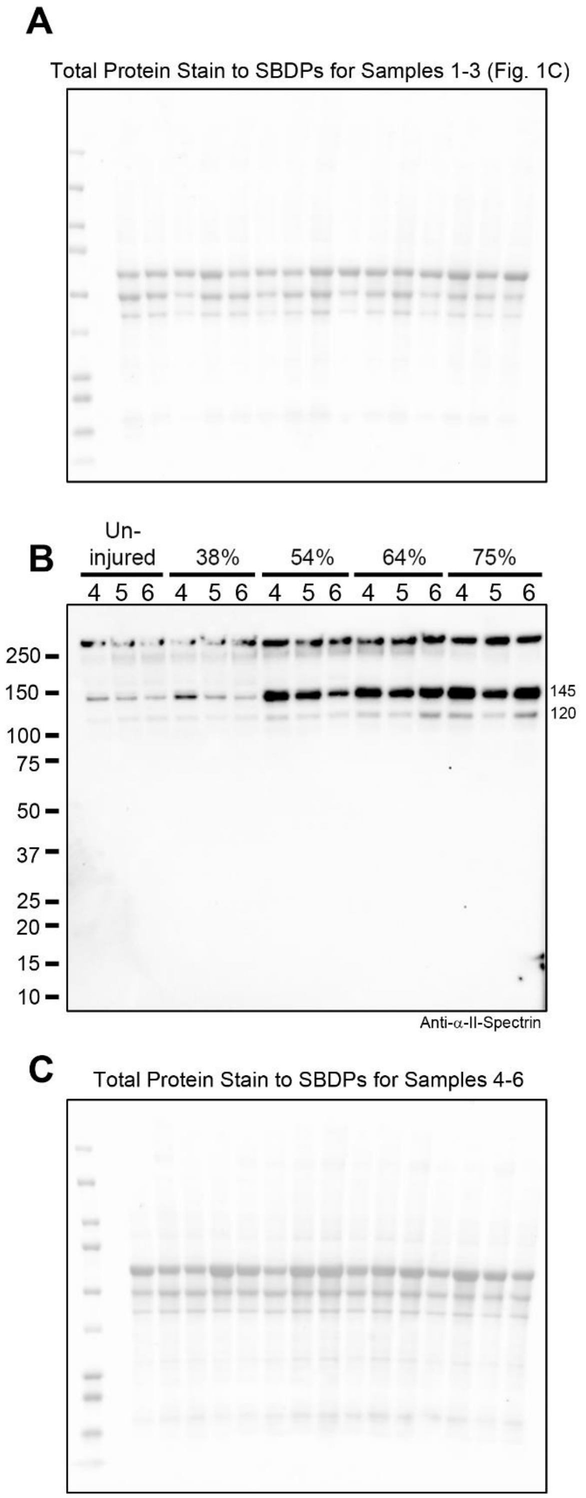

Fig. S2

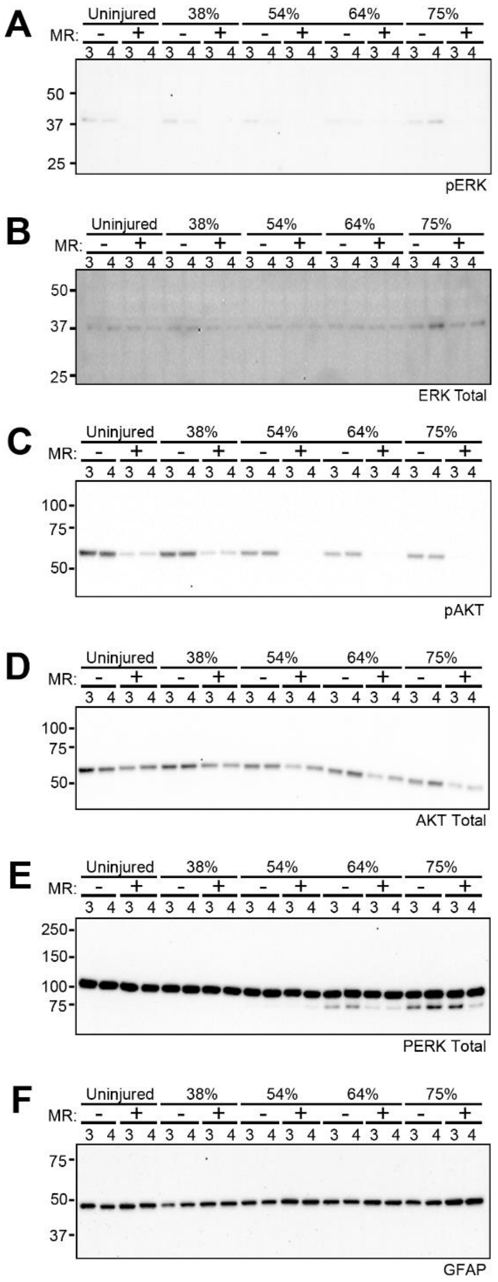
A
Uninjured
38%
54%
64%
75%
MR:
50
37
25
pERK
B
ERK Total
C
100
75
pAKT
D
AKT Total
E
250
150
PERK Total
F
GFAP

Fig. S3

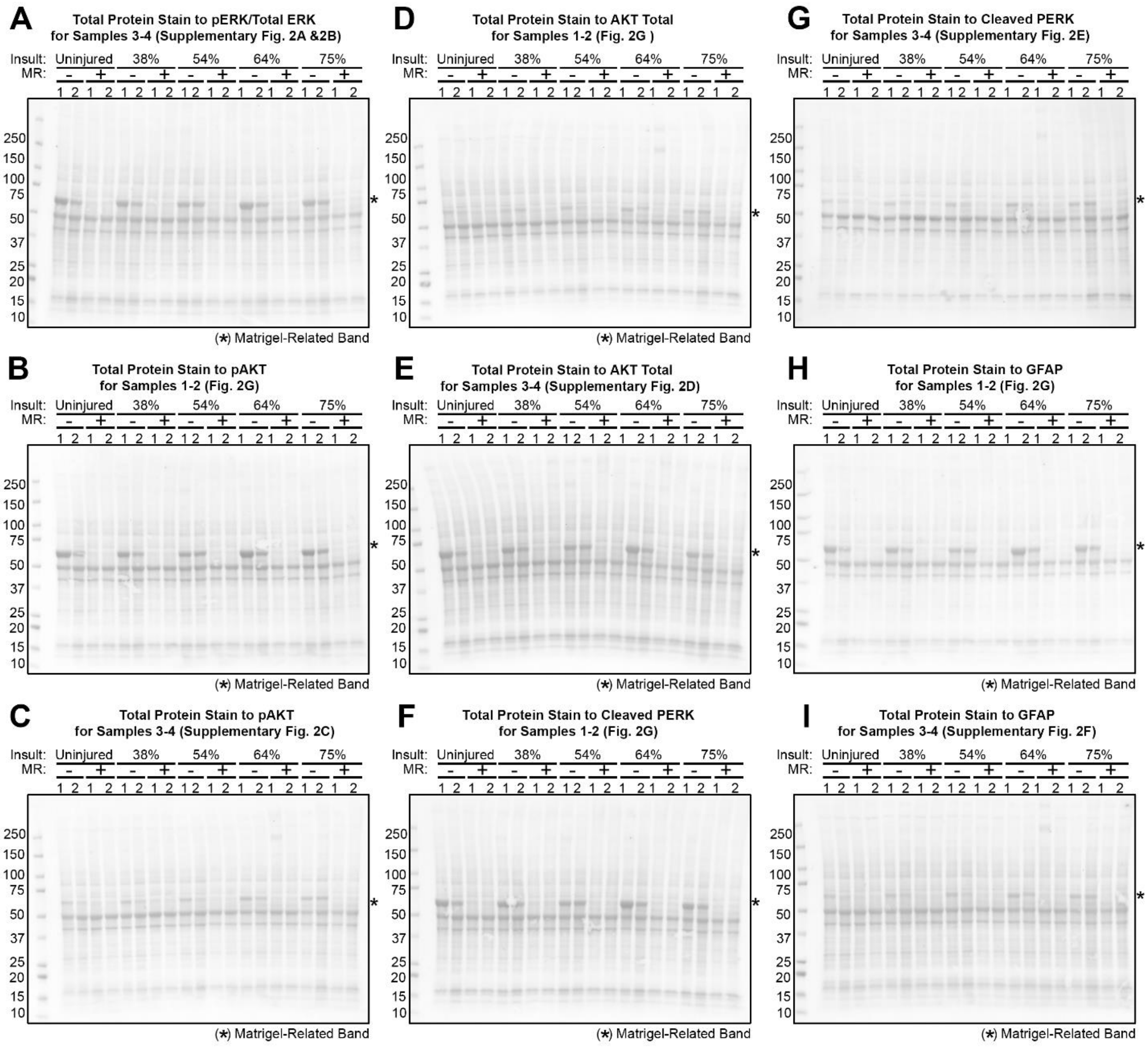
A
Total Protein Stain to pERK/Total ERK
for Samples 3-4 (Supplementary Fig. 2A &2B)
D
Total Protein Stain to AKT Total
for Samples 1-2 (Fig. 2G)
G
Total Protein Stain to Cleaved PERK
for Samples 3-4 (Supplementary Fig. 2E)
B
Total Protein Stain to pAKT
for Samples 1-2 (Fig. 2G)
E
Total Protein Stain to AKT Total
for Samples 3-4 (Supplementary Fig. 2D)
H
Total Protein Stain to GFAP
for Samples 1-2 (Fig. 2G)
C
Total Protein Stain to pAKT
for Samples 3-4 (Supplementary Fig. 2C)
F
Total Protein Stain to Cleaved PERK
for Samples 1-2 (Fig. 2G)
I
Total Protein Stain to GFAP
for Samples 3-4 (Supplementary Fig. 2F)
Insult: Uninjured 38% 54% 64% 75%
MR: - + - + - + - + - +
250
150
100
75
50
37
25
20
15
10
*
(*) Matrigel-Related Band

Fig. S4

A

Total Protein Stain to SBDPs for Samples 1-4 (Fig. 3D)

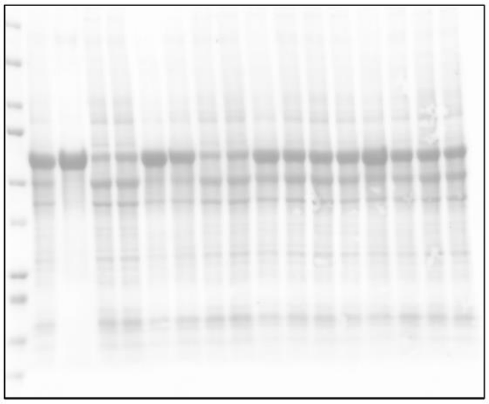

B

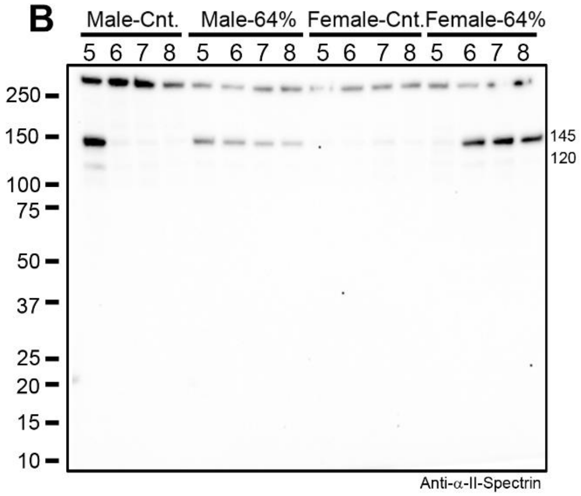

C

Total Protein Stain to SBDPs for Samples 5-8

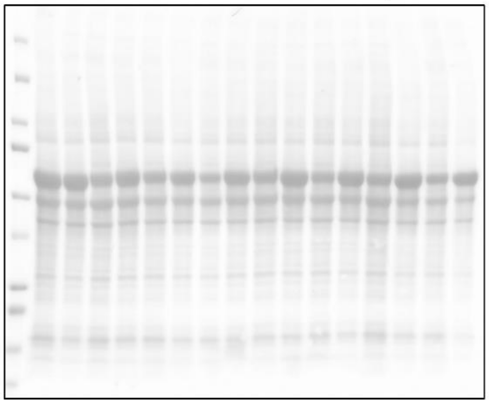

Fig. S5

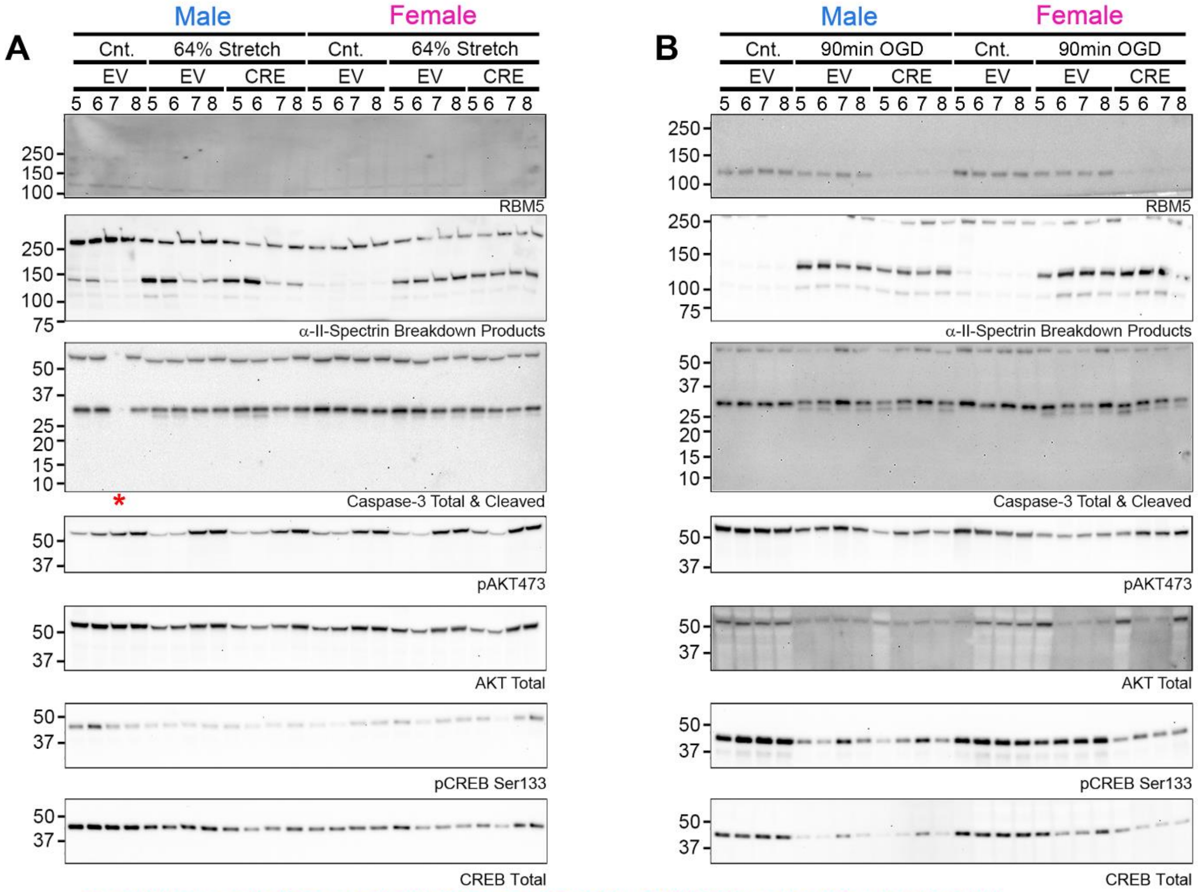

* = Empty Lane for Caspase-3-Total Sample **Male-Cnt.-EV-#7** (Due to Sample Unavailability)

Fig. S6

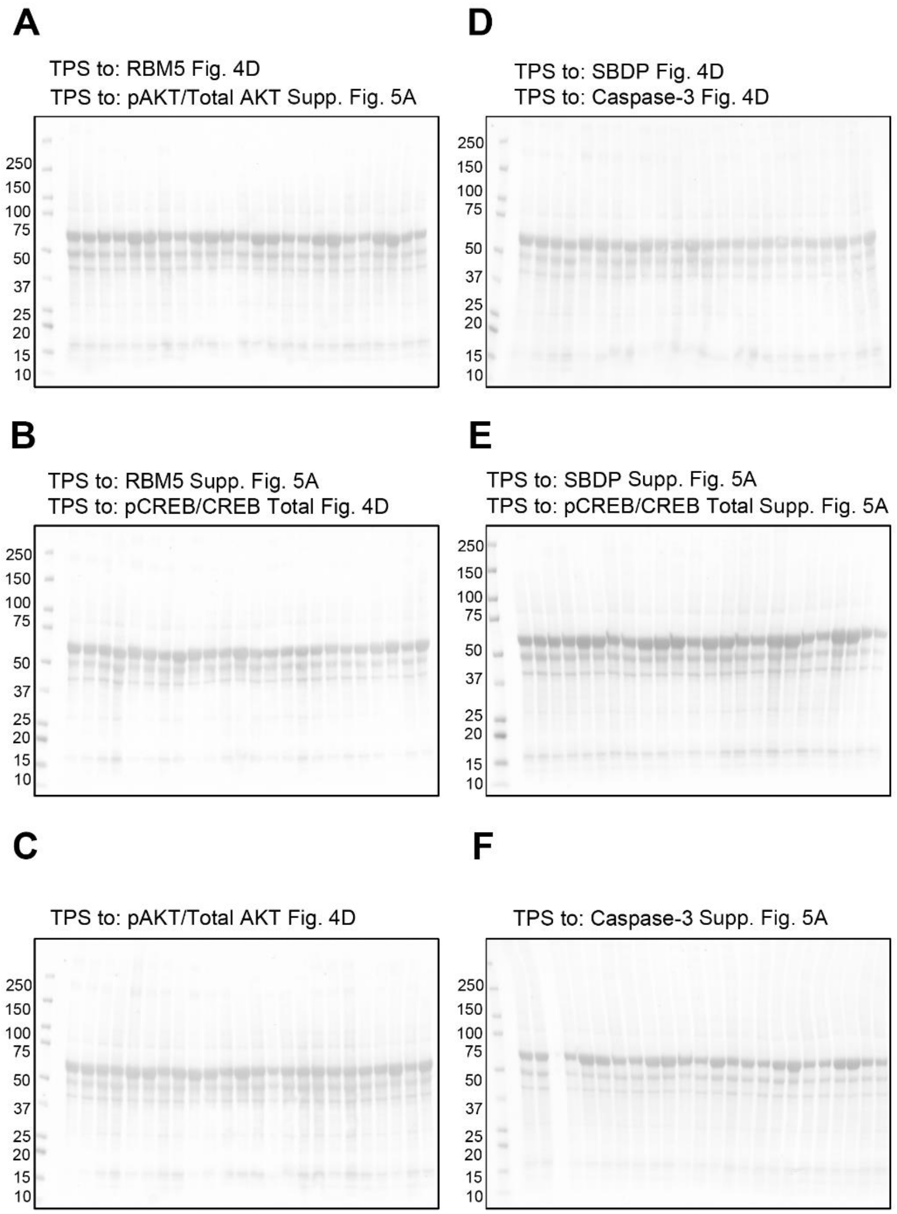
A
TPS to: RBM5 Fig. 4D
TPS to: pAKT/Total AKT Supp. Fig. 5A
B
TPS to: RBM5 Supp. Fig. 5A
TPS to: pCREB/CREB Total Fig. 4D
C
TPS to: pAKT/Total AKT Fig. 4D
D
TPS to: SBDP Fig. 4D
TPS to: Caspase-3 Fig. 4D
E
TPS to: SBDP Supp. Fig. 5A
TPS to: pCREB/CREB Total Supp. Fig. 5A
F
TPS to: Caspase-3 Supp. Fig. 5A
250
150
100
75
50
37
25
20
15
10

Fig. S7

A

TPS to: RBM5 Fig. 5E
TPS to: Caspase-3 Fig. 5E

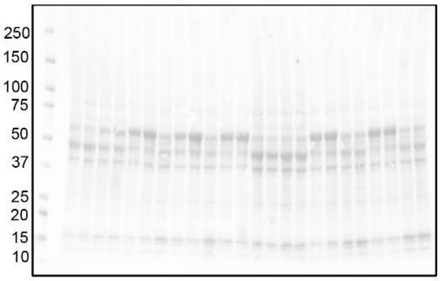

B

TPS to: RBM5 Supp. Fig. 5B
TPS to: Caspase-3 Supp. Fig. 5B

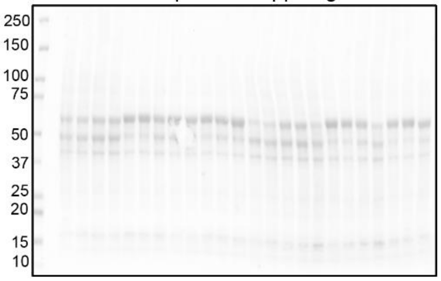

C

TPS to: SBDP Fig. 5E
TPS to: pAKT/Total AKT Fig. 5E

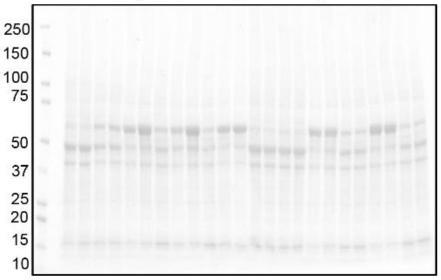

D

TPS to: SBDP Supp. Fig. 5B
TPS to: pAKT/Total AKT Supp. Fig. 5B

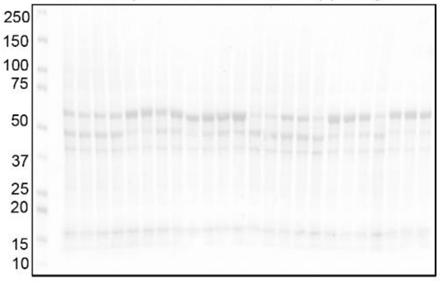

E

TPS to: pCREB/Total CREB Fig. 5E

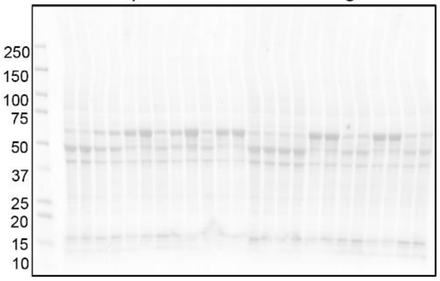

F

TPS to: pCREB/Total CREB Supp. Fig. 5B

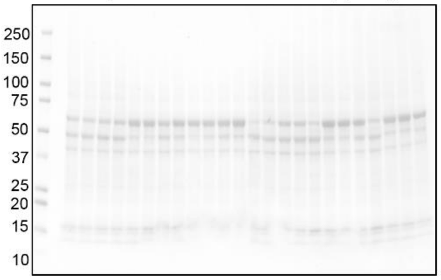

Fig. S8

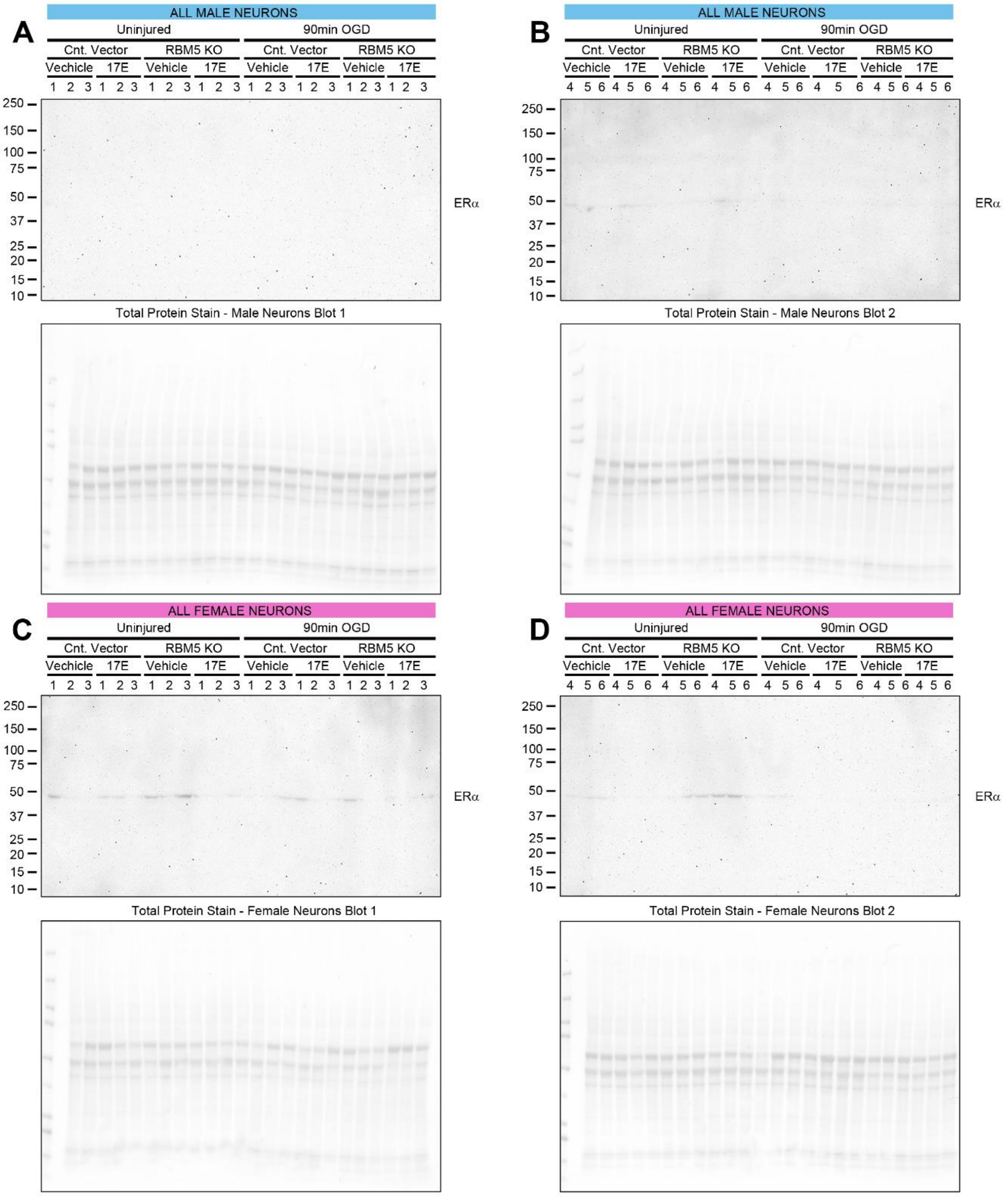
A
B
C
D
ALL MALE NEURONS
ALL FEMALE NEURONS
Uninjured
90min OGD
Cnt. Vector
RBM5 KO
Vechicle
Vehicle
17E
ERα
250
150
100
75
50
37
25
20
15
10
Total Protein Stain - Male Neurons Blot 1
Total Protein Stain - Male Neurons Blot 2
Total Protein Stain - Female Neurons Blot 1
Total Protein Stain - Female Neurons Blot 2

Fig. S9

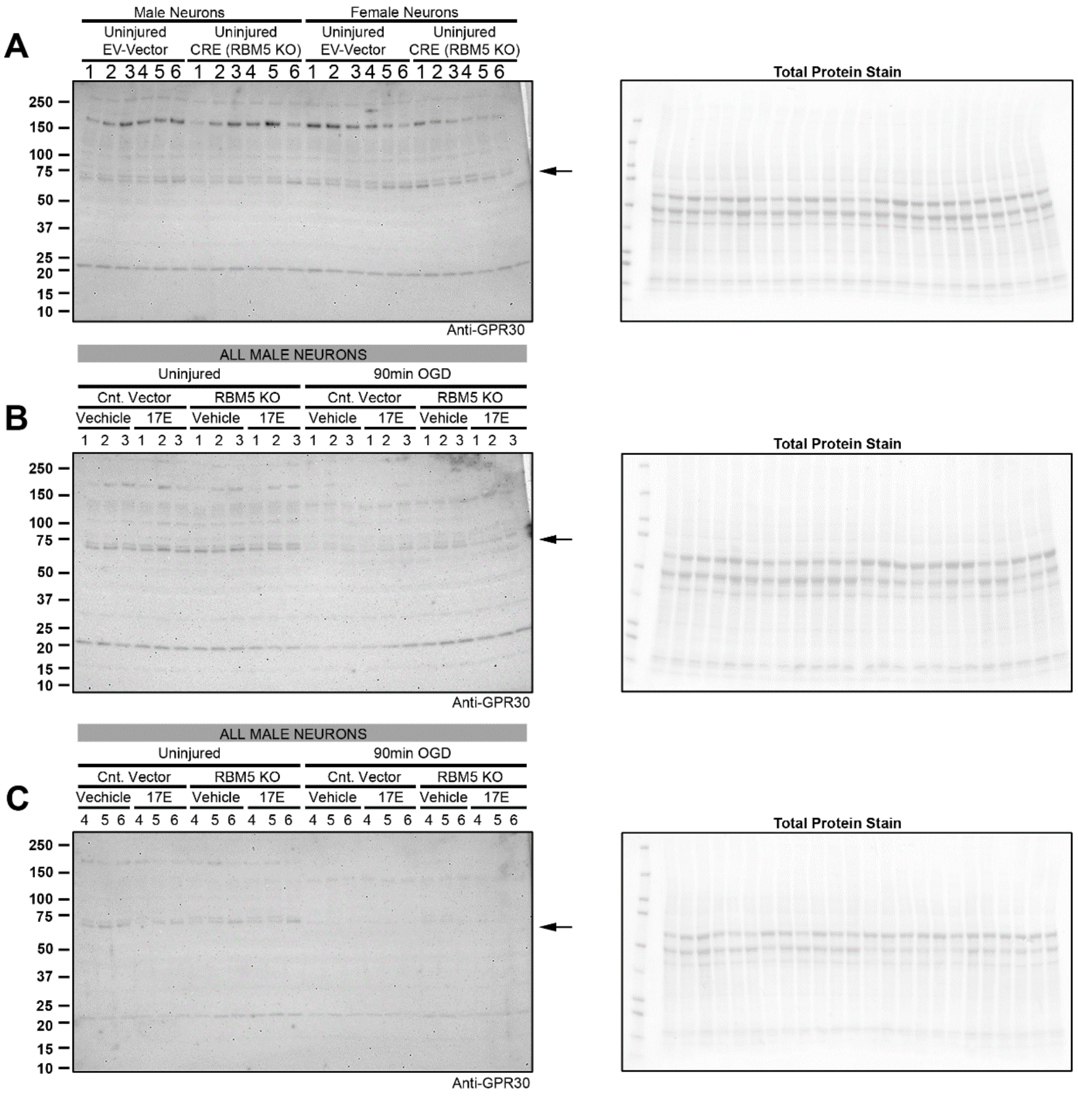
A
Male Neurons
Female Neurons
Uninjured EV-Vector
Uninjured CRE (RBM5 KO)
Total Protein Stain
Anti-GPR30
250
150
100
75
50
37
25
20
15
10
B
ALL MALE NEURONS
Uninjured
90min OGD
Cnt. Vector
RBM5 KO
Vechicle
Vehicle
17E
Total Protein Stain
Anti-GPR30
C
ALL MALE NEURONS
Uninjured
90min OGD
Cnt. Vector
RBM5 KO
Vechicle
Vehicle
17E
Total Protein Stain
Anti-GPR30

Fig. S10

A

Total Protein Stain to SBDP Blot in Fig 6D

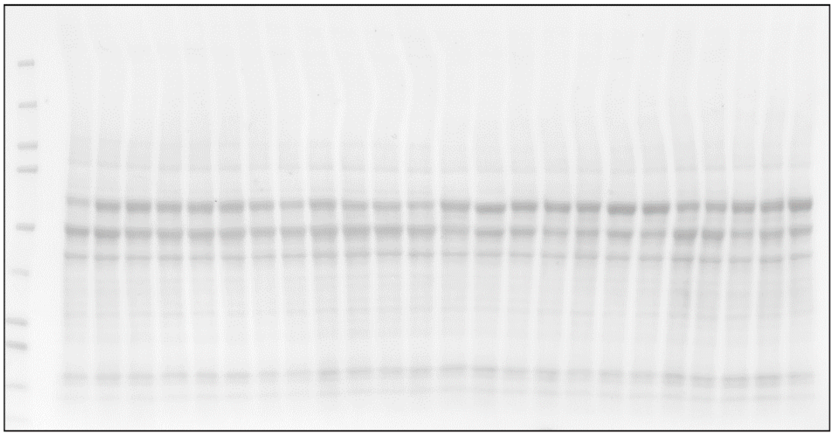

(All Male Samples)

B

Total Protein Stain to SBDP Blot in Fig. 6E

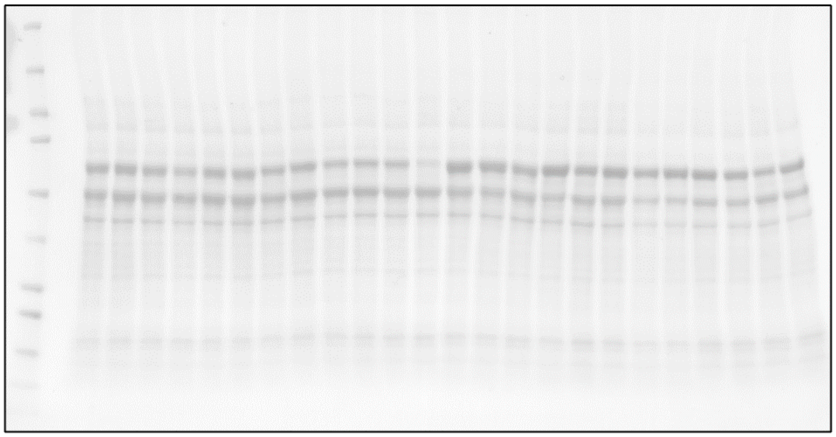

(All Female Samples)

Fig. S11

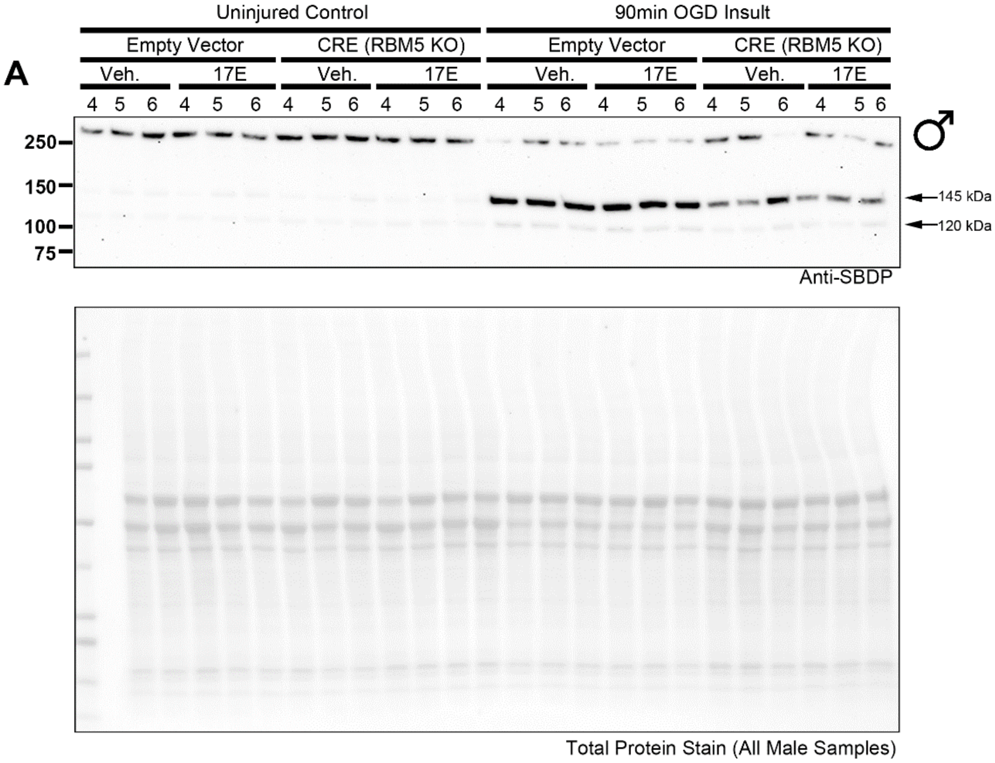
Uninjured Control
90min OGD Insult
Empty Vector
CRE (RBM5 KO)
Empty Vector
CRE (RBM5 KO)
Veh.
17E
Veh.
17E
Veh.
17E
Veh.
17E
4 5 6 4 5 6 4 5 6 4 5 6 4 5 6 4 5 6 4 5 6 4 5 6
250
150
100
75
♂
145 kDa
120 kDa
Anti-SBDP
Total Protein Stain (All Male Samples)

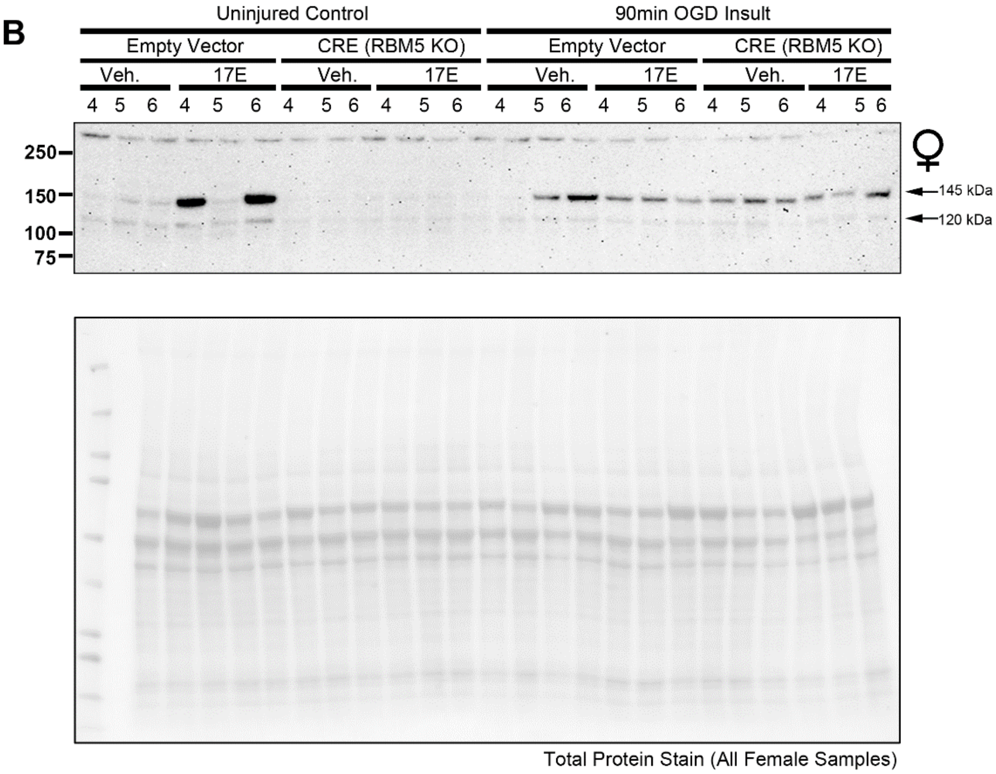
Uninjured Control
90min OGD Insult
Empty Vector
CRE (RBM5 KO)
Empty Vector
CRE (RBM5 KO)
Veh.
17E
Veh.
17E
Veh.
17E
Veh.
17E
4 5 6 4 5 6 4 5 6 4 5 6 4 5 6 4 5 6 4 5 6 4 5 6
250
150
100
75
♀
145 kDa
120 kDa
Total Protein Stain (All Female Samples)

Fig. S12

A

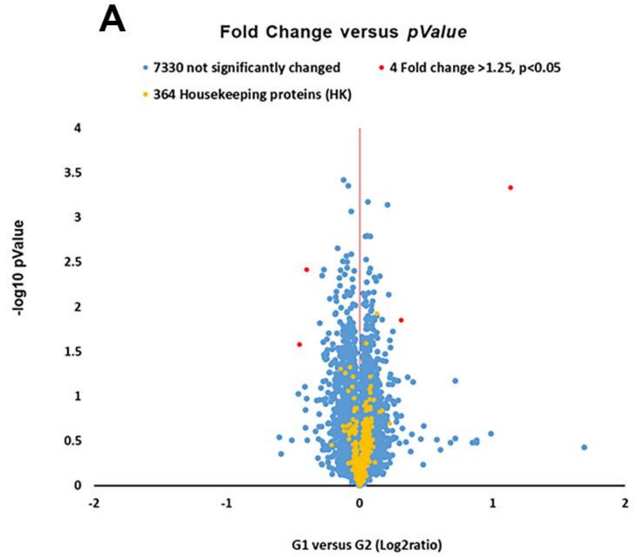
Fold Change versus pValue
7330 not significantly changed
4 Fold change >1.25, p<0.05
364 Housekeeping proteins (HK)
-log10 pValue
G1 versus G2 (Log2ratio)

B

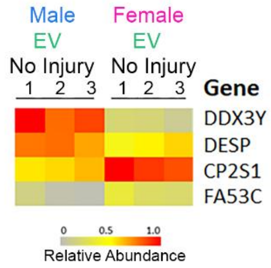
Male
EV
No Injury
Female
EV
No Injury
1 2 3
1 2 3
Gene
DDX3Y
DESP
CP2S1
FA53C
0 0.5 1.0
Relative Abundance

Fig. S13

TMT Analysis Neuron Cultures

A

SRY-Confirmed

Isolation 1:
Male Purity 100%
Female Purity 100%
Isolation 2:
Male Purity 100%
Female Purity 100%
Isolation 3:
Male Purity 100%
Female Purity 100%

B

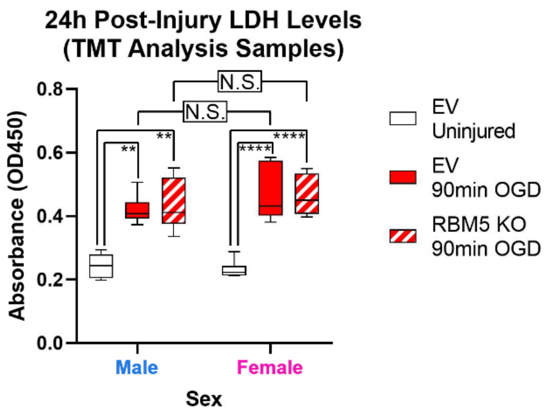

Fig. S14

Uncropped Blots (Fig. 2)

pERK (Fig. 2C)

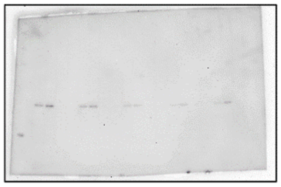

pERK (Supplementary Fig. 2A)

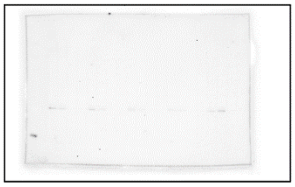

Total ERK (Fig. 2E)

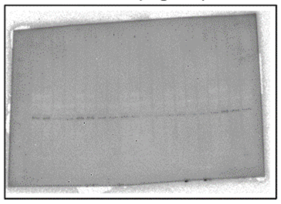

Total ERK (Supplementary Fig. 2B)

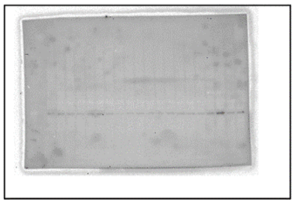

pAKT (Fig. 2G)

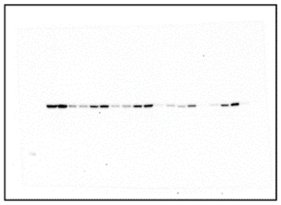

pAKT (Supplementary Fig. 2C)

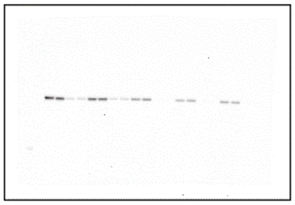

Total AKT (Fig. 2G)

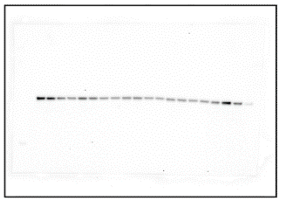

Total AKT (Supplementary Fig. 2D)

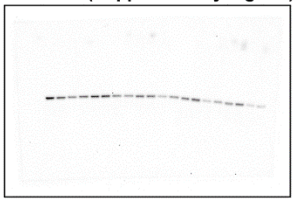

PERK (Fig. 2G)

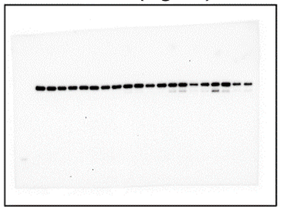

PERK (Supplementary Fig. 2E)

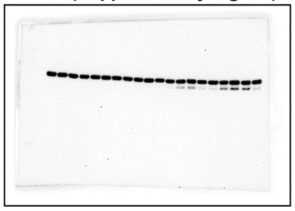

GFAP (Fig. 2G)

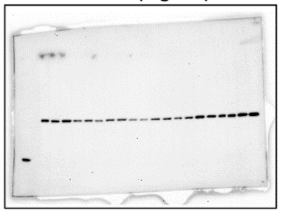

GFAP (Supplementary Fig. 2F)

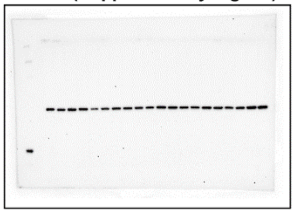

Fig. S15

Uncropped Blots (Fig. 4 and Fig. 5)

RBM5 (Fig. 4D)

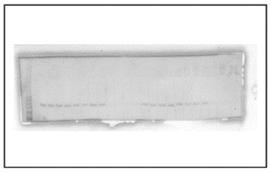

RBM5 (Supplementary Fig. 5A)

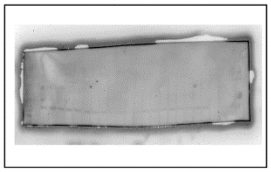

RBM5 (Fig. 5E)

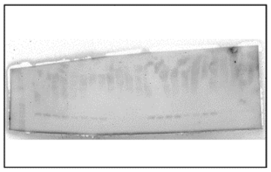

RBM5 (Supplementary Fig. 5B)

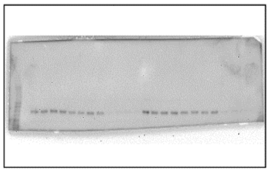

SBDP (Fig. 4D)

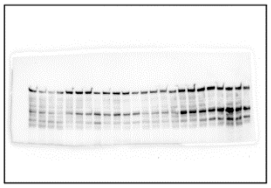

SBDP (Supplementary Fig. 5A)

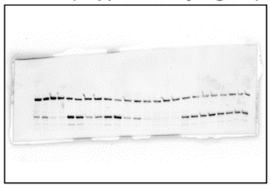

SBDP (Fig. 5E)

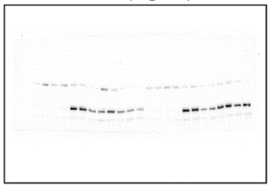

SBDP (Supplementary Fig. 5B)

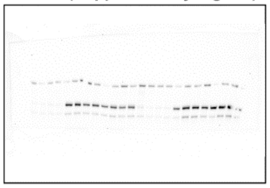

Caspase-3 (Fig. 4D)

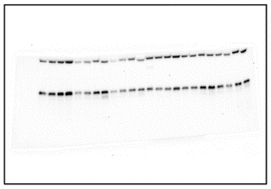

Caspase-3 (Supplementary Fig. 5A)

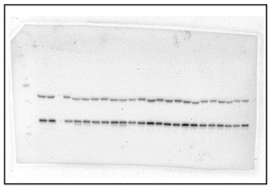

Caspase-3 (Fig. 5E)

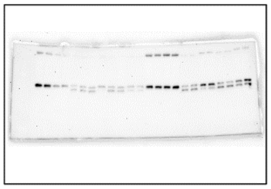

Caspase-3 (Supplementary Fig. 5B)

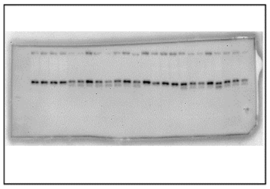

pAKT (Fig. 4D)

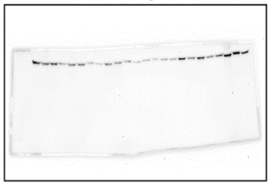

pAKT (Supplementary Fig. 5A)

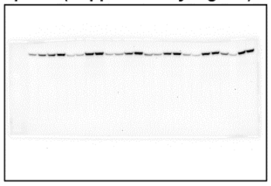

pAKT (Fig. 5E)

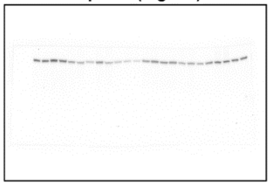

pAKT (Supplementary Fig. 5B)

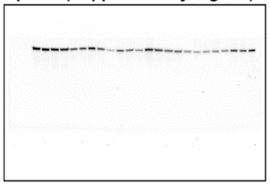

Total AKT (Fig. 4D)

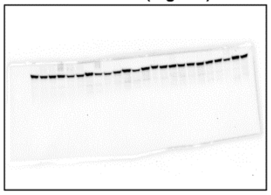

Total AKT (Supplementary Fig. 5A)

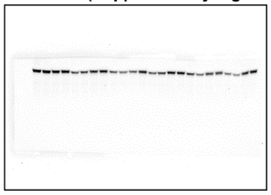

Total AKT (Fig. 5E)

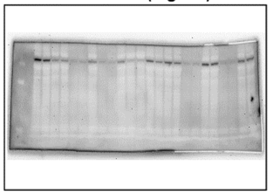

Total AKT (Supplementary Fig. 5B)

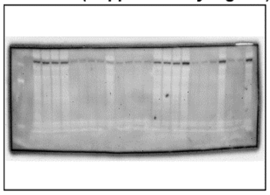

pCREB (Fig. 4D)

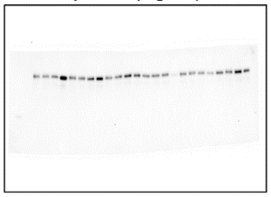

pCREB (Supplementary Fig. 5A)

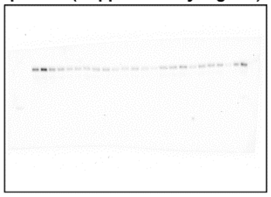

pCREB (Fig. 5E)

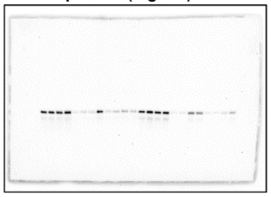

pCREB (Supplementary Fig. 5B)

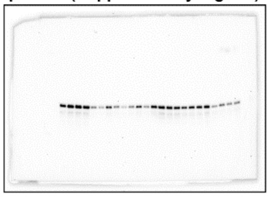

Total CREB (Fig. 4D)

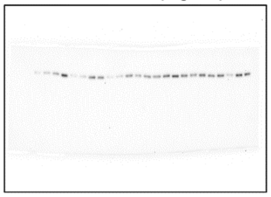

Total CREB (Supplementary Fig. 5A)

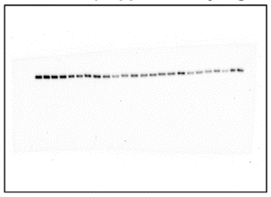

Total CREB (Fig. 5E)

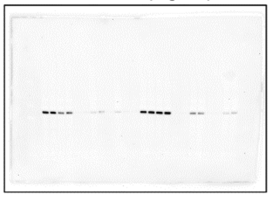

Total CREB (Supplementary Fig. 5B)

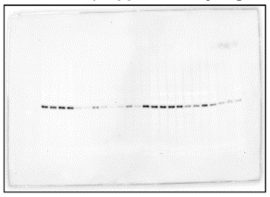

Fig. S16

Uncropped Blots (Fig. 6)

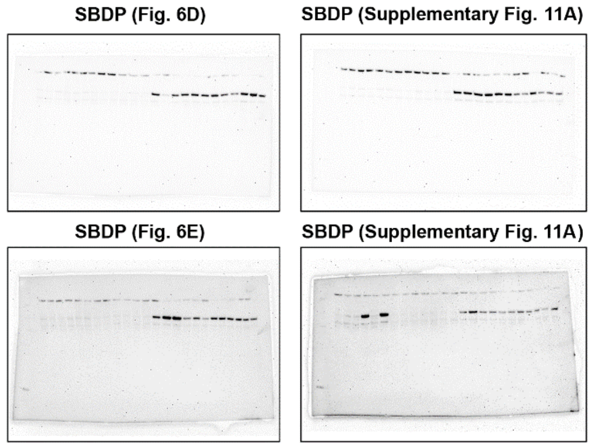

Fig. S17

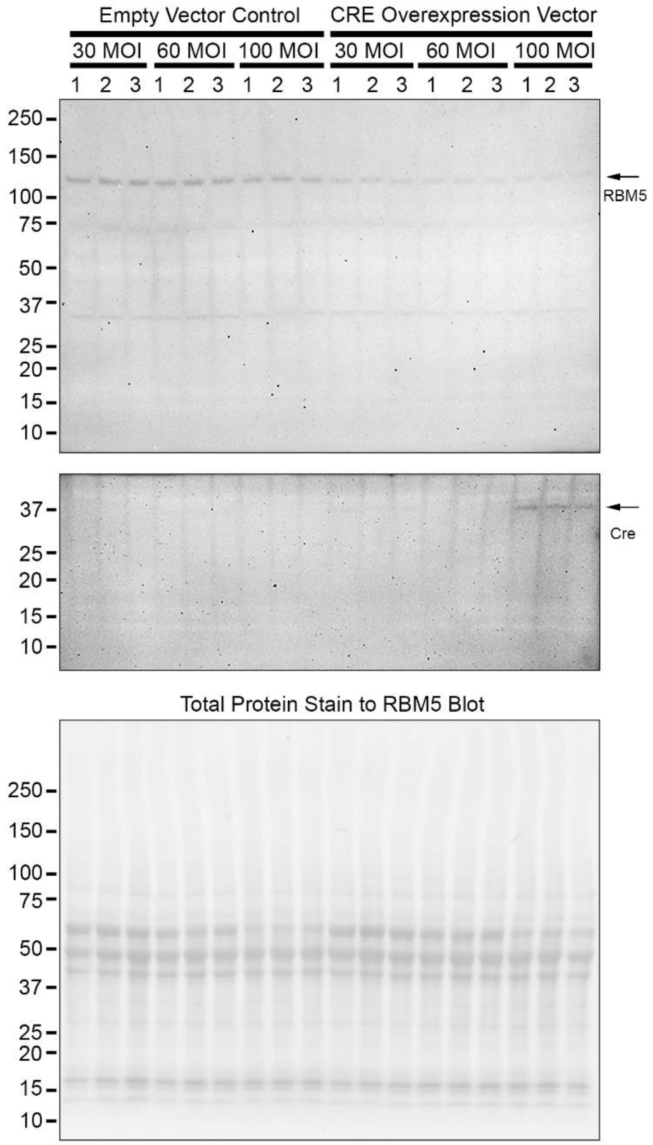
Empty Vector Control
CRE Overexpression Vector
30 MOI
60 MOI
100 MOI
30 MOI
60 MOI
100 MOI
1 2 3 1 2 3 1 2 3 1 2 3 1 2 3 1 2 3
250
150
100
75
50
37
25
20
15
10
RBM5
37
25
20
15
10
Cre
Total Protein Stain to RBM5 Blot
250
150
100
75
50
37
25
20
15
10

Supplementary Tables 1-10

Statistical Tests & Outcomes

Table 1 Statistical Results: Matrigel Removal Protocol Protein Targets (2-WAY-ANOVA)

Figure Panel	Target	Passed Normality Test	Gaussian Distribution	Aligned Rank Transformation Of Data (ARTool)	Factor 1: Matrigel Removal	Factor 2: Insult Severity	Interaction Term
Fig. 2 Panel D	Phospho-ERK	NO **(p=0.0001)**	NO	YES	**p<0.0001**	**p=0.0002**	**p<0.0001**
Fig. 2 Panel F	Total ERK	YES (p=0.0510)	YES	NO	p=0.2162	**p=0.0002**	p=0.6792
Fig. 2 Panel H	Phospho-AKT	YES (p=0.1000)	YES	NO	**p<0.0001**	**p<0.0001**	**p<0.0001**
Fig. 2 Panel I	Total AKT	YES (p=0.1000)	YES	NO	**p<0.0001**	**p=0.0143**	p=0.2594
Fig. 2 Panel J	Cleaved PERK	NO **(p<0.0001)**	NO	YES	**p<0.0001**	**p<0.0001**	**p=0.0014**
Fig. 2 Panel K	Total GFAP	NO **(p=0.0475)**	NO	YES	**p=0.0018**	**p=0.0019**	p=0.3957

Table 2 Statistical Results: Mechanical Stretch WT Neurons Peak Pressure, LDH, & Protein Targets (Unpaired t-test and 2-WAY-ANOVA)

Figure Panel	Target	Passed Normality Test	Gaussian Distribution	Aligned Rank Transformation Of Data (ARTool)	Factor 1: Injury	Factor 2: Sex	Interaction Term
Fig. 3 Panel A	Well Peak Pressure (Injured)	NO **(p=0.0013)**	NO	YES	p=0.2331	**N/A**	**N/A**
Fig. 3 Panel C	LDH Levels	NO **(p=0.0149)**	NO	YES	**p<0.0001**	p=0.0924	p=0.7451
Fig. 3 Panel E	SBDP 145 Levels	NO **(p=0.0035)**	NO	YES	**p<0.0007**	p=0.1265	p=0.0804

Table 3 Statistical Results: Mechanical Stretch Floxed Neurons Peak Pressure & LDH (3-WAY-ANOVA)

Figure Panel	Target	Passed Normality Test	Gaussian Distribution	Aligned Rank Transformation Of Data (ARTool)	Factor 1: Injury	Factor 2: Sex	Factor 3 Genotype
Fig. 4 Panel A	Well Peak Pressure	NO **(p<0.0001)**	NO	YES	**p<0.0001**	**p<0.0001**	p=0.0805
Fig. 4 Panel C	LDH Levels	YES (p=0.1000)	YES	NO	**p<0.0001**	p=0.9112	p=0.2148
Cont'd	**Interaction Term 1 Injury x Sex**	**Interaction Term 2 Injury x Genotype**	**Interaction Term 3 Sex x Genotype**	**Interaction Term 4 Genotype x Sex x Injury**			
Fig. 4 Panel A	**p<0.0001**	p=0.0805	**p=0.0080**	**p=0.0080**			
Fig. 4 Panel C	p=0.9752	p=0.1525	p=0.4924	p=0.4338			

Table 4 Statistical Results: Mechanical Stretch Floxed Neurons Protein Targets (2-WAY-ANOVA)

Figure Panel	Target	Passed Normality Test	Gaussian Distribution	Aligned Rank Transformation Of Data (ARTool)	Factor 1: Vector/Injury	Factor 2: Sex	Interaction Term
Fig. 4 Panel E	RBM5 Levels	YES (p=0.1000)	YES	NO	**p<0.0001**	**p=0.0004**	**p<0.0195**
Fig. 4 Panel F	SBDP 145 Levels	NO **(p=0.0128)**	NO	YES	**p<0.0001**	**p=0.0019**	**p=0.0015**
Fig. 4 Panel G	pAKT473 Levels	NO **(p=0.0072)**	NO	YES	p=0.2503	p=0.8461	p=0.1482
Fig. 4 Panel H	pCREB133 Levels	YES (p=0.1000)	YES	NO	p=0.2395	p=0.5732	**p=0.0456**

Table 5 Statistical Results: OGD LDH WT Neurons (2-WAY-ANOVA)

Figure Panel	Target	Passed Normality Test	Gaussian Distribution	Aligned Rank Transformation Of Data (ARTool)	Factor 1: Injury	Factor 2: Sex	Interaction Term
Fig. 5 Panel A	LDH Levels	NO **(p=0.0124)**	NO	YES	**p<0.0001**	p=0.1323	**p=0.0043**

Table 6 Statistical Results: OGD LDH RBM5 Floxed Neurons (3-WAY-ANOVA)

Figure Panel	Target	Passed Normality Test	Gaussian Distribution	Aligned Rank Transformation Of Data (ARTool)	Factor 1: Injury	Factor 2: Sex	Factor 3 Genotype
Fig. 5 Panel C	LDH Levels	YES (p=0.1000)	YES	NO	**p<0.0001**	**p=0.0170**	p=0.6416
Cont'd	**Interaction Term 1 Injury x Sex**	**Interaction Term 2 Injury x Genotype**	**Interaction Term 3 Sex x Genotype**	**Interaction Term 4 Genotype x Sex x Injury**			
Fig. 5 Panel C	p=0.8277	p=0.1087	p=0.2146	p=0.8339			

Table 7 Statistical Results: OGD Protein Targets Floxed Neurons (2-WAY-ANOVA)

Figure Panel	Target	Passed Normality Test	Gaussian Distribution	Aligned Rank Transformation Of Data (ARTool)	Factor 1: Vector/Injury	Factor 2: Sex	Interaction Term
Fig. 5 Panel F	RBM5 Levels	NO **(p=0.0017)**	NO	YES	**p<0.0001**	p=0.6510	**p=0.0013**
Fig. 5 Panel G	SBDP 145 Levels	NO **(p=0.0015)**	NO	YES	**p<0.0001**	**p=0.0004**	**p=0.0018**
Fig. 5 Panel H	SBDP 120 Levels	YES (p=0.1000)	YES	NO	**p<0.0001**	**p=0.0078**	p=0.3917
Fig. 5 Panel I	Caspase 3 Fragment	NO **(p=0.0209)**	NO	YES	**p<0.0001**	**p=0.0135**	**p=0.0072**
Fig. 5 Panel J	pAKT473 Levels	YES (p=0.1000)	YES	NO	**p<0.0001**	p=0.2200	**p<0.0001**
Fig. 5 Panel K	pCREB133 Levels	YES (p=0.1000)	YES	NO	**p<0.0001**	p=0.1194	p=0.1803

Table 8 Statistical Results: OGD Floxed Neurons & Estradiol Treatment LDH (3-WAY-ANOVA)

Figure Panel	Target	Passed Normality Test	Gaussian Distribution	Aligned Rank Transformation Of Data (ARTool)	Factor 1: Injury	Factor 2: Drug	Factor 3 Genotype
Fig. 6 Panel A	LDH Levels	NO **(p<0.0209)**	NO	YES	**p<0.0001**	p=0.1700	p=0.1990
Fig. 6 Panel B	LDH Levels	NO **(p<0.0440)**	NO	YES	**p<0.0001**	**p=0.0090**	p=0.1297
Cont'd	**Interaction Term 1 Injury x Drug**	**Interaction Term 2 Injury x Genotype**	**Interaction Term 3 Drug x Genotype**	**Interaction Term 4 Genotype x Drug x Injury**			
Fig. 6 Panel A	p=0.2480	p=0.0568	p=0.9849	p=0.8944			
Fig. 6 Panel B	**p=0.0033**	p=0.0842	p=0.9534	p=0.4760			

Table 9 Statistical Results: OGD Floxed Neurons & Estradiol Treatment SBDP Targets (3-WAY-ANOVA)

Figure Panel	Target	Passed Normality Test	Gaussian Distribution	Aligned Rank Transformation Of Data (ARTool)	Factor 1: Injury	Factor 2: Drug	Factor 3 Genotype
Fig. 6 Panel F	SBDP Levels 145kDa	NO **(p<0.0001)**	NO	YES	**p<0.0001**	**p=0.0537**	**p=0.0025**
Fig. 6 Panel G	SBDP Levels 145kDa	NO **(p=0.0029)**	NO	YES	**p<0.0001**	p=0.4425	p=0.5196
Fig. 6 Panel H	SBDP Levels 120kDa	YES **(**p=0.1000**)**	YES	NO	**p<0.0001**	p=0.0669	p=0.9995
Fig. 6 Panel I	SBDP Levels 120kDa	YES **(**p=0.1000**)**	YES	NO	p=0.3085	p=0.6086	p=0.1120
Cont'd	**Interaction Term 1 Injury x Drug**	**Interaction Term 2 Injury x Genotype**	**Interaction Term 3 Drug x Genotype**	**Interaction Term 4 Genotype x Drug x Injury**			
Fig. 6 Panel F	p=0.0593	**p=0.0029**	p=0.1900	p=0.1257			
Fig. 6 Panel G	p=0.0225	p=0.1762	p=0.2485	p=0.0190			
Fig. 6 Panel H	p=0.2414	p=0.6696	p=0.3096	p=0.2025			
Fig. 6 Panel I	p=0.3705	**p=0.0314**	**p=0.0372**	**p=0.0019**			

Table 10 Statistical Results: OGD Floxed Neurons LDH for TMT Study (2-WAY-ANOVA)

Figure Panel	Target	Passed Normality Test	Gaussian Distribution	Aligned Rank Transformation Of Data (ARTool)	Factor 1: Vector/Injury	Factor 2: Sex	Interaction Term
Supplementary Figure	LDH Levels	NO (p=0.0055)	NO	YES	**p<0.0001**	p=0.2547	p=0.4646

Supplementary Table 11

Embryo Number	Sex Determination Made Visually	Actual Sex (SRY Genotyping)	Incorrect Assignment (**X**)	Embryo Culture Isolation Date
1	Female	Female		4.2.21
2	Female	Female		
3	Male	Male		
4	Female	Female		
5	Male	Male		
6	Female	Female		
7	Male	Male		
8	Male	Male		
9	Male	Male		
10	Male	Male		
11	Female	Female		
12	Male	Female	**X**	
13	Male	Male		
14	Male	Male		
15	Male	Male		
16	Male	Male		
17	Female	Female		
18	Female	Female		
19	Female	Female		
20	Female	Female		
21	Female	Female		
22	Male	Male		
23	Male	Male		
24	Male	Male		
25	Male	Male		4.30.21
26	Female	Male	**X**	Out of Protocol
27	Male	Male		(Below 80%)
28	Male	Male		
29	Female	Female		
30	Male	Male		
31	Male	Male		
32	Male	Male		
33	Male	Male		
34	Male	Female	**X**	
35	Male	Female	**X**	
36	Female	Female		
37	Male	Female	**X**	
38	Female	Female		
39	Female	Female		
40	Male	Male		
41	Female	Female		5.3.21
42	Female	Male	**X**	

43	Male	Male		
44	Female	Female		
45	Male	Male		
46	Male	Male		
47	Female	Female		
48	Female	Female		
49	Female	Female		
50	Female	Female		
51	Female	Female		
52	Male	Male		
53	Female	Female		
54	Male	Male		
55	Female	Female		
56	Male	Male		5.7.21
57	Male	Male		
58	Male	Male		
59	Male	Male		
60	Female	Female		
61	Female	Female		
62	Female	Male	**X**	
63	Male	Male		
64	Female	Female		
65	Male	Male		
66	Female	Female		
67	Female	Male	**X**	5.21.21
68	Male	Male		
69	Male	Male		
70	Female	Female		
71	Female	Female		
72	Male	Male		
73	Male	Male		
74	Male	Male		
75	Female	Female		
76	Female	Female		
77	Male	Male		
78	Female	Female		
79	Female	Female		
80	Male	Male		
81	Female	Female		
82	Male	Male		
83	Female	Female		
84	Female	Female		
85	Male	Male		
86	Male	Male		
87	Male	Male		
88	Female	Female		4.21.22

89	Female	Female		
90	Male	Female	X	
91	Male	Male		
92	Male	Male		
93	Female	Female		
94	Male	Male		
95	Male	Male		
96	Female	Female		
97	Male	Male		
98	Female	Female		
99	Female	Male	X	
100	Male	Male		5.12.22
101	Female	Female		
102	Female	Female		
103	Male	Male		
104	Female	Female		
105	Male	Male		
106	Male	Male		
107	Male	Male		
108	Male	Male		
109	Male	Male		
110	Female	Female		
111	Male	Male		
112	Male	Male		
113	Male	Male		
114	Female	Female		
115	Male	Male		
116	Female	Female		
117	Female	Female		6.13.22
118	Female	Female		
119	Male	Male		
120	Male	Male		
121	Male	Male		
122	Male	Male		
123	Female	Female		
124	Male	Male		
125	Male	Male		
126	Female	Female		
127	Female	Female		
128	Female	Female		
129	Male	Female	X	6.17.22
130	Male	Male		
131	Male	Male		
132	Female	Female		
133	Female	Female		
134	Female	Female		

135	Female	Female		
136	Female	Female		
137	Female	Female		
138	Male	Male		
139	Female	Female		
140	Female	Female		
141	Male	Male		
142	Female	Female		
143	Female	Female		
144	Male	Male		
145	Male	Male		
146	Male	Male		7.5.22
147	Female	Female		
148	Female	Female		
149	Female	Female		
150	Male	Male		
151	Male	Male		
152	Female	Female		
153	Female	Female		
154	Male	Male		
155	Male	Male		
156	Male	Male		
157	Female	Female		
158	Female	Female		
159	Male	Male		
160	Male	Male		
161	Female	Female		
162	Female	Female		
163	Male	Male		
164	Female	Female		
165	Male	Male		
166	Female	Female		
167	Female	Female		
167	Male	Male		
168	Male	Male		7.8.22
169	Female	Female		
170	Male	Male		
171	Female	Female		
172	Female	Female		
173	Female	Female		
174	Male	Male		
175	Male	Male		7.11.22
176	Male	Male		
177	Male	Male		
178	Male	Male		
179	Male	Male		

180	Female	Female		
181	Male	Male		
182	Male	Male		
183	Female	Female		
184	Female	Female		
185	Male	Male		
186	Female	Female		
187	Female	Female		
188	Male	Male		
189	Male	Male		
190	Female	Female		
191	Male	Male		
192	Female	Female		
193	Male	Male		
194	Male	Male		
195	Male	Male		
196	Female	Female		
197	Male	Male		
198	Female	Female		
199	Male	Male		
200	Female	Female		
201	Female	Female		
202	Female	Female		
203	Female	Female		8.30.22
204	Female	Female		
205	Female	Female		
206	Female	Female		
207	Male	Male		
208	Female	Male	X	
209	Female	Female		
210	Female	Female		
211	Female	Female		
212	Male	Male		
213	Male	Male		
214	Female	Female		
215	Male	Male		
216	Female	Female		
217	Male	Male		
218	Male	Male		
219	Male	Male		
220	Male	Male		
221	Male	Male		
222	Female	Female		
223	Male	Male		
224	Male	Male		
225	Female	Female		

225	Female	Female		
226	Female	Female		
227	Female	Female		
228	Female	Female		
229	Female	Female		
230	Female	Female		
231	Female	Female		
232	Female	Female		
233	Female	Female		9.12.22
234	Male	Male		
235	Male	Male		
236	Female	Female		
237	Male	Male		
238	Male	Male		
239	Male	Male		
240	Female	Female		
241	Female	Female		
242	Male	Male		
243	Male	Male		
244	Female	Female		
245	Male	Male		
246	Female	Female		
247	Female	Female		
248	Male	Male		
249	Female	Female		
250	Male	Male		
251	Male	Male		
252	Male	Male		
253	Female	Female		
254	Male	Male		10.10.22
255	Male	Male		
256	Female	Female		
257	Female	Female		
258	Male	Male		
259	Male	Male		
260	Male	Male		
261	Female	Female		
262	Female	Female		
263	Female	Female		
264	Male	Male		
265	Female	Female		
266	Female	Female		
267	Male	Male		
268	Male	Male		10.13.22
269	Female	Female		
270	Female	Female		

271	Male	Male		
272	Male	Male		
273	Female	Female		
274	Male	Male		
275	Female	Female		
276	Male	Male		
277	Male	Male		
278	Female	Female		
279	Female	Female		
280	Female	Female		
281	Male	Male		
282	Male	Male		
283	Male	Male		
284	Female	Female		
285	Female	Female		
286	Female	Female		
287	Female	Female		
288	Male	Male		
289	Female	Female		
290	Male	Male		
291	Female	Female		
292	Female	Female		
293	Male	Male		
294	Male	Male		
295	Male	Male		
296	Male	Male		
297	Male	Male		
298	Male	Male		
299	Male	Male		
300	Male	Male		
301	Female	Female		
302	Male	Male		
303	Female	Female		
304	Male	Male		10.26.22
305	Female	Female		
306	Male	Male		
307	Female	Female		
308	Female	Female		
309	Male	Male		
310	Male	Male		
311	Male	Male		
312	Male	Male		
313	Male	Male		
314	Female	Female		
315	Male	Male		
316	Male	Male		

317	Male	Male		
318	Male	Male		
319	Female	Female		
320	Male	Male		
321	Female	Female		
322	Male	Male		
323	Female	Female		
324	Female	Female		
325	Female	Female		11.15.22
326	Female	Female		
327	Male	Male		
328	Female	Female		
329	Male	Male		
330	Male	Male		
331	Male	Male		
332	Male	Male		
333	Female	Female		
334	Female	Female		
335	Male	Male		
336	Male	Male		
337	Male	Male		
338	Male	Male		
339	Female	Female		
340	Female	Female		
341	Female	Female		
342	Male	Male		
343	Female	Female		
344	Male	Male		
345	Male	Male		
346	Male	Male		11.21.22
347	Female	Female		
348	Female	Female		
349	Male	Male		
350	Male	Male		
351	Male	Male		
352	Male	Male		
353	Male	Male		
354	Male	Male		
355	Female	Female		
356	Male	Male		
357	Female	Female		
358	Male	Male		
359	Female	Female		
360	Male	Male		
361	Male	Male		
362	Female	Female		

363	Male	Male		
364	Male	Male		
365	Female	Female		
366	Female	Female		
367	Female	Female		
368	Female	Female		
369	Female	Female		11.28.22
370	Female	Female		
371	Male	Male		
372	Female	Female		
373	Female	Female		
374	Male	Male		
375	Male	Male		
376	Female	Female		
377	Male	Male		
378	Male	Male		
379	Male	Male		
380	Male	Male		
381	Male	Male		
382	Female	Female		
383	Female	Female		
384	Female	Female		
385	Male	Male		
386	Male	Male		
387	Female	Female		
388	Male	Male		
389	Male	Male		
390	Female	Female		
391	Male	Male		
392	Male	Male		
393	Female	Female		
394	Male	Male		
395	Female	Female		
396	Female	Female		
397	Male	Male		
398	Male			12.7.22
399	Female			
400	Female			
401	Female			
402	Male			
403	Male			
404	Male			
405	Female			
406	Female			
407	Female			
408	Male			

409	Male			
410	Male			
411	Male			
412	Male			
413	Male			
414	Male			
415	Male			
416	Female			
417	Female			3.14.23
418	Female			**Male Dishes Lost**
419	Female			**(Technical Issue)**
420	Female			
421	Female			
422	Female			
423	Female			
424	Female			
425	Female			
426	Female			
427	Female			3.21.23
428	Female			
429	Male			
430	Female			
431	Female			
432	Female			
433	Female			
434	Male			
435	Male			
436	Male			
437	Male			
438	Male			
439	Female			
440	Female			
441	Male			
442	Male			
443	Female			
444	Female			
445	Male			
446	Male			
447	Male			
448	Female			
449	Male			
450	Male			
451	Female			
452	Female			
453	Male			
454	Male			

456	Male			
457	Female			
458	Female			
459	Male			
460	Female			
461	Female			
462	Male			
463	Female			
464	Male			
465	Female			
466	Female			
467	Male			

12 Embryos Incorrectly Assigned out of 467 Total Embryos = **97.4% Success Rate to Appropriately Assign Sex**
